# Tracking Inter‐ and Intraspecific Bacterial Competition in Pairwise Assays Using a Transient Fluorescent Dye

**DOI:** 10.1002/ece3.73395

**Published:** 2026-04-07

**Authors:** Julius Hoffmann, Ole Johannsen, Lutz Becks

**Affiliations:** ^1^ Aquatic Ecology and Evolution University of Konstanz Konstanz Germany

**Keywords:** bacterial competition, competitiveness, imaging flow‐cytometry, pairwise competition assays, relative fitness, transient fluorescent dyes

## Abstract

Testing inter‐ and intraspecific competition is essential for understanding the processes that maintain diversity within communities and populations. Pairwise growth assays are well‐established methods to study the outcome of competition between bacterial genotypes or species. However, distinguishing bacterial competitors in coculture remains a major challenge due to their small cell size, similar cell morphology, and often large population sizes. Current approaches are either labour intensive and require prior morphological and sequence‐based characterisation or genetic modification of the bacteria. We present a simple assay that tracks the relative abundances of two competing bacterial species or genotypes of the same species through transient staining of one competitor with a fluorescent dye. The use of a transient dye relieves prior genetic characterisation or modification. Our approach uses a sample‐dependent threshold evaluation for the fluorescent intensity, enabling the quantification of bacterial frequencies over generations. The method provides high accuracy for tracking competing genotypes and species. The outcome of competition often depends on frequency‐dependent fitness effects and/or environmental variables. Therefore, our method tests across different initial abundances and can also assess the role of a biotic interaction (presence/absence of a predator) on the outcome of competition. Our method presents a simple and high‐throughput assay for competition. Absence of genetic modifications and applicability to little characterised competitors make it particularly useful for assays involving bacterial isolates from evolving populations.

## Graphical abstract

Understanding how competition maintains diversity is central to ecology and evolution. We introduce a simple, high‐throughput assay to quantify relative abundances of bacterial types during pairwise competition using a transient fluorescent dye. Absence of genetic modifications and applicability to little characterised competitors make it particularly useful for assays involving bacterial isolates from evolving populations.

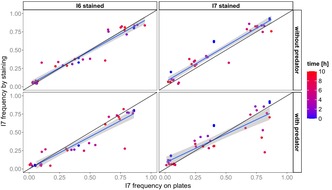

## 1 Introduction

The quantification of competition between organisms is a central goal in biology to better understand the maintenance of inter‐ and intraspecific diversity and evolutionary outcomes (Antonovics and Kareiva 1988; Foster and Bell 2012). In ecology, the strength of interspecific competition determines the coexistence of species and thus community diversity (Chesson 2000; Freilich et al. 2011; Menge and Sutherland 1976). In evolutionary biology and population genetics, intraspecific competition between genotypes drives evolutionary change (Dykhuizen 1990). Understanding competition is also essential for biotechnological applications (Kinkel et al. 2011; Merritt and Edwards 2004). For instance, when microbial communities are optimised for agriculture or wastewater treatment (Fried et al. 2000; Guingo et al. 1998), it is crucial to identify which environmental factors (e.g., nutrient supply, temperature, trophic interactions) promote the desired competitive outcomes (Burian et al. 2022; Hiltunen et al. 2008; Klitgord and Segrè 2010; Minty et al. 2013).

In both intra‐ and interspecific competition, the outcome is determined by relative fitness: the more competitive type contributes more offspring to subsequent generations, replacing the less competitive type(s) over time. The rate of this replacement can be used to quantify competitiveness. However, competitive hierarchies often shift with environmental changes (Menéndez‐Serra et al. 2022). If the environment changes continuously, dominant competitors may also change dynamically (Hutchinson 1961; Wei et al. 2017; Zhao et al. 2023). Beyond abiotic variation, the biotic environment can strongly influence competition. For example, predators can decisively alter the outcome between prey types (Chase et al. 2002; Meyer and Kassen 2007). Trade‐offs between reproduction and defense, or frequency‐dependent effects, can also reverse or stabilise competitive hierarchies, leading to coexistence under certain conditions (Yoshida et al. 2004, 2007). Hence, to understand competition comprehensively, it must be quantified across diverse environmental contexts.

In bacterial competition experiments, several approaches have been developed to track relative abundances in mixed cultures. Traditional fluorescent protein–based methods distinguish competitors using constitutively expressed fluorescent markers detected by flow cytometry, microscopy, or spectrometry (Gallet et al. 2012; Schlechter et al. 2021). While these approaches provide single‐cell resolution, they require stable genetic modification of each competitor, which can be technically challenging, resource‐intensive, and may alter cellular physiology or competitive fitness. Such constraints limit their use when working with environmental isolates, evolutionarily dynamic populations, or non‐model species where genetic transformation is inefficient or undesirable.

Sequencing‐based approaches (e.g., 16S rRNA profiling, metabarcode tracking) offer a marker‐free alternative and are powerful for studying complex microbial communities (Harvey et al. 2021; Howells et al. 2025). However, they rely on prior genomic information and costly DNA extraction, library preparation and bioinformatic analysis. Moreover, sequencing provides endpoint or delayed information, making it less suited for tracking rapid competition dynamics in short‐term, high‐replicate laboratory assays.

Our transient dye–based approach addresses these limitations by combining the speed and simplicity of fluorescence‐based detection with the flexibility and noninvasiveness of sequencing‐free assays. By transiently staining one competitor, cocultures can be quantified without genetic modification or genomic data, enabling application to diverse bacterial isolates and short‐term ecological experiments. This is particularly advantageous when competitors differ in their evolutionary history, plasmid stability, or transformation capacity–conditions common in experimental ecology and biotechnology.

A key feature of our method is the use of a receiver–operator characteristic (ROC)‐based threshold to classify stained versus unstained cells objectively. This data‐driven cutoff adapts to the empirical fluorescence overlap between populations, overcoming the subjectivity of fixed‐threshold or visual classification used in conventional fluorescence‐based methods. As a result, our framework extends high‐throughput pairwise competition assays to systems where genetic labeling or sequencing‐based quantification are impractical, providing a rapid, generalisable and low‐cost avenue to study ecological interactions.

## 2 Material and Methods

*Pseudomonas fluorescens* genotypes I6 and I7 were isolated from a previous long‐term evolution experiment (Kičiatovas et al. 2026). Briefly, *P. fluorescens* SBW 25 was cocultured with the ciliate predator *Tetrahymena thermophila* strain 1630/1 U (CCAP) in culture flasks containing 6 mL 5% King's B (KB) medium, maintained at 28°C under 1% serial transfer every seven days. Genotypes I6 and I7 were isolated from the LTEE by plating a thawed sample from a freeze‐stored subsample from the LTEE at transfer 90 on PPY growth agar. These plates were grown at 28°C and 10 colonies were randomly picked. These colonies were grown in 1.5 mL PPY at 28°C for 24 h and then freeze‐stored (−80°C) in 85% Glycerol. For the present study we used isolates I6 and I7. For the species competition, clonal populations of *P. fluorescens* strain SBW25 and *Escherichia coli* strain ATCC 11303 (American Type Culture Collection) were used. Both strains were freeze‐stored in 85% Glycerol.

### 2.1 Competition Assay

Before the competition assays, bacterial stocks were revived from the glycerol stocks and preconditioned in 5% King's B (KB) medium. Of each competitor one population was stained using the transient fluorescent dye 10× CellBriteFix 640 solution, then stained and unstained competitors were combined in cocultures at three ratios (0.1, 0.5, 0.9) (see staining procedure and preparation of cocultures). For all assays, the mono‐ and cocultures were transferred to wells of a deep‐well microtiter plate where each condition (stained competitor, starting relative abundance, predator presence) was replicated three times. If predators were included in the assay they were handled as described below and also added to the microtiter plate. Competition assays were incubated for 10 h at 20°C and sampled every 2 h. During this time the microtiter plate was kept inside an Opentrons OT‐2 (Opentrons Labworks Inc., Long Island City, United States) pipetting robot equipped with an HEPA filter to provide a sterile environment. For each sampling time point, two samples were taken from each microcosm. One set of samples was automatically taken and fixed with Formaldehyde (2% final concentration). These samples were analysed using the AMNIS ImageStream MkII (Cytek Biosciences B.V., Amsterdam, The Netherlands) imaging flow‐cytometer and their optical density (OD, 600 nm) was measured using a BioTek Epoch 2 spectrophotometer (Agilent, Santa Clara, United States) to track population dynamics. The second set of samples was serially diluted and plated on PPY agar plates to enumerate the competitions.

### 2.2 Staining Procedure and Competition Assay

Two days prior to the competition assay, bacterial stocks were revived from freeze‐storage by incubating 6 μL of stock in 6 mL of PPY for 24 h at 28°C under shaking. Then 100 μL of culture were given to fresh 10 mL 5% KB medium and cultures were pre‐conditioned for further 24 h at 28°C with shaking. Bacterial densities of the pre‐conditioned culture were measured (OD600) and adjusted to similar levels. For staining, 9 mL of each culture was centrifuged (10 min, 3740 × ***g***), the supernatant discarded, and the cells were resuspended in 3 mL Phosphate Buffered Saline (PBS). The cultures were then split between two 2 mL centrifuge tubes, which were washed in PBS (centrifuged 10 min, 10,000 × ***g***, resuspended in 1.5 mL PBS). After a second centrifugation (10 min, 10,000 × ***g***), the supernatant was discarded. To one tube per bacterial strain, we added 400 μL of a 10× CellBriteFix 640 solution, while 400 μL of M9 salt solution was added to the other. The estimated cell concentrations during staining were: I7 = 1.07*10^9^ cells/mL, I6 = 1.51*10^9^ cells/mL, *P.fluorescens* = 6.54*10^9^ cells/mL, *E.coli* = 5.62*10^9^ cells/mL. All tubes were then incubated in the dark for 30 min at 28°C., then washed three times (i.e., removing the supernatant after centrifugation; 10 min, 10,000 × ***g***) and resuspended in 1.5 mL PBS. After the final round of centrifugation, cells were resuspended in 1.5 mL M9 salt solution and each culture was given to 8.5 mL 5% KB medium. From these four monocultures six cocultures containing both competitors were prepared: the stained competitor was always paired with an unstained competitor and each pairing was prepared in three ratios (0.1, 0.5, 0.9). 100 μL of each coculture was taken to estimate initial relative abundances of both competitors by plating on growth agar. To start the competition assays 800 μL from the cocultures were given to three wells (six if predator dependence of competition is tested) on a deep‐well microtiter plate. Previously, these wells had either been filled with 500 μL 5% KB medium or with 500 μL of predator culture (see below). To ensure equal treatment of stained and unstained cells, unstained cells underwent the same washing and incubation steps as stained cells.

### 2.3 Preparing Predators for the Competition

A culture of *Tetrahymena thermophila* CCAP 1630/1 U was serially maintained in Proteose Peptone Yeast extract (PPY) medium under axenic conditions at 28°C. Three to five days ahead of the competition assay, a new subculture of the ciliates was started (200 μL of old culture in 50 mL PPY at 28°C). On the day of the experiment, the predator culture was washed twice with M9 salt solution (centrifugation 10 min, 600 × ***g*** at 10°C; resuspension in 20 mL salt solution). After the second centrifugation, the supernatant was discarded and cells resuspended in 15 mL fresh salt solution. To estimate cell density, 0.5 mL samples were taken, diluted 1:9 in 4.5 mL 11% Lugol's solution (500 μL 100% Lugol in 4 mL M9 solution), and *Tetrahymena* cells in 10 μL of sample were enumerated using a Neubauer haemocytometer. This culture was subsequently diluted with 5% KB medium to reach a cell concentration of 30,000 cells/mL. Finally, 500 μL of this predator culture were then given to the appropriate wells on the deep‐well microtiter plate for the competition assay.

### 2.4 Co‐Culture Evaluation Imaging Flow‐Cytometry

Fixed samples from the staining test and competition assays were analysed using an AMNIS ImageStream imaging flow‐cytometer. For each sample, 2000 bacterial cells were acquired using the 60× objective (max. acquisition time 8 min). Laser intensity was set to: 405 nm: 120 mW, 488 nm: 50 mW, 642 nm: 0.5 mW, SSC: 0.8 mW. Bacterial cells were distinguished from all objects via two gates: (1) Cells, including those fluorescently stained, were separated from other objects in the sample based on fluorescence intensity channel 11 (fluorescence of dye) and channel 2 (autofluorescence of cells) by retaining objects within the following coordinates (Ch11, Ch2): (0, 0), (1000, 0), (14147935.62, 0), (14147935.62, 18258919.88), (129532.65, 11248522.44), (5402.53, 22932.84), (0, 1481.21); (2) bacterial cells were separated from ciliates based on size (area channel 9 (brightfield), values retained: 2–150). The raw data acquired by the ImageStream were processed via the proprietary IDEAS 6.2 software. Then channel 11 fluorescence intensity values for each bacterial cell were extracted. The values from unstained and stained monocultures were used to determine optimal thresholds for classifying stained and unstained cells. For competition assays, these thresholds were then applied to fluorescence intensity values from cocultures to determine the relative abundance of competitors.

### 2.5 Fluorescence Thresholds by Receiver‐Operator Characteristic Curves

Optimal fluorescence thresholds were determined by combining single‐cell fluorescence data from stained and unstained monocultures. An algorithm tested all possible fluorescence values between the observed minimum and maximum as potential thresholds, classifying each cell as stained (≥ threshold) or unstained (< threshold). Classification accuracy was assessed by comparing each cell's classification to its known origin. Matches were considered correct classifications. For each threshold, True Positive Rate (TPR) and False Positive Rate (FPR) were calculated. Receiver Operating Characteristic curves (ROC) were generated by plotting FPR against TPR, and the optimal threshold was defined as the point closest to the top‐left corner–maximising both TPR and FPR. The performance of these optimal thresholds was further validated by testing their performance on a second set of stained and unstained controls. All operations were performed using Python (version 3.13.3). ROCs were generated using the roc_curve function from the scikit‐learn API (version 1.6).

### 2.6 Coculture Evaluation on Growth Agar

To evaluate competitor relative abundances determined by fluorescence intensity, samples from the competition assay were also plated on growth agar (50% PPY). For initial relative abundances the six pre‐mixed cocultures were used, while during the experiment, samples were directly taken from cultures of the competition assay. For each sample, we plated a dilution series on agar (1:10^3.79^, 1:10^4.50^, 1:10^5.19^, 1:10^5.89^, 1:10^6.59^, 1:10^7.29^). After 24 h of growth at 28°C, the number of bacterial colonies and the abundance of both competitors were counted. Only plates with 30 to 300 colonies were evaluated. For each coculture only competitor relative abundance from the plate with the highest number of colonies was used for further analysis.

### 2.7 Statistical Analysis

The comparison between competitor frequencies based on the fluorescence staining and growth agar was assessed using the Pearson's coefficient of correlation, examining the overall correlation as well as that for each stained competitor and, if applicable, predator presence. Competitor frequency dynamics over time were analysed by estimating the slopes of frequency trajectories throughout the competition assays with a linear mixed model (LME, R package lmerTest). The frequency of one competitor was the dependent variable explained by the fixed effects: time, initial abundance of the competitor, stained competitor, predator presence and interactions between time and each of the three other fixed effects. We included a random intercept to account for random variation between replicate competition cocultures. For each combination of initial abundance, stained competitor and predator presence, the temporal trend was estimated using the *emtrends* function from R package emmeans. Using a linear model (LM), we assessed whether the carrying capacity of cocultures was dependent on the starting frequencies of competitors. Carrying capacity was taken as the highest OD value measured per coculture and then we averaged across the three replicates per experimental condition. In the LM, mean carrying capacity was explained by the fixed effects initial abundance, stained competitor and predator presence. Estimated Marginal Means of carrying capacity for each treatment combination were then computed using the *emmeans* function (R package emmeans).

### 2.8 Media Composition

5% King's B (KB) medium: 1 g Proteose peptone N°3, 0.5 mL 85% Glycerol and 11.28 g 5× M9 salts per 1 L deionised water. 100% Proteose Peptone Yeast extract medium (PPY): 20 g Bacteriological peptone MC024 (Lab M Limited), 2.5 g yeast extract (Lab M Limited, CAS: 8013‐01‐2) per 1 L deionised water.

## 3 Results

### 3.1 Staining by the Fluorescent Dye

We tested different volumes of the fluorescent dye to determine the optimal amount needed to stain bacterial populations. Monocultures of the *P. fluorescens* isolate I7 were stained with four different volumes (400 μL, 300 μL, 200 μL, 100 μL) of a 10× solution of the fluorescence dye and were incubated, alongside two unstained controls, for 10 h. All cultures were sampled every 2 h to measure fluorescence intensity per cell using an imaging flow‐cytometer to assay staining success. For this we calculated the fluorescence thresholds for the classification of stained and unstained cells and evaluated their performance using ROCs. ROCs were used to determine thresholds that separated between a given stained sample (dye volume, time point) and an unstained control. For each dye volume we determined thresholds based on the time points 0 and 5 (10 h), and a general threshold based on all time points and compared their classification performance (Table S1, Figures S1–S3). We assessed performance using several metrics: *accuracy* of the classification i.e., the proportion of cells that were correctly classified, the True Positive Rate (TPR) i.e., the proportion of cells that were correctly classified as stained, the True Negative Rate (TNR) i.e., the proportion of cells that were correctly classified as unstained, and the *average* between TPR and TNR. Across all dye concentrations, a general threshold allowed a higher *accuracy* in classifying stained and unstained cells (all = 0.993, TP0 = 0.581, TP5 = 0.584). Second, we tested threshold performance at different dye volumes using only the general thresholds (Table S2). The best classification was achieved with 400 μL dye (threshold = 375.915, *accuracy* = 0.995, *average* = 0.995).

**FIGURE 1:**
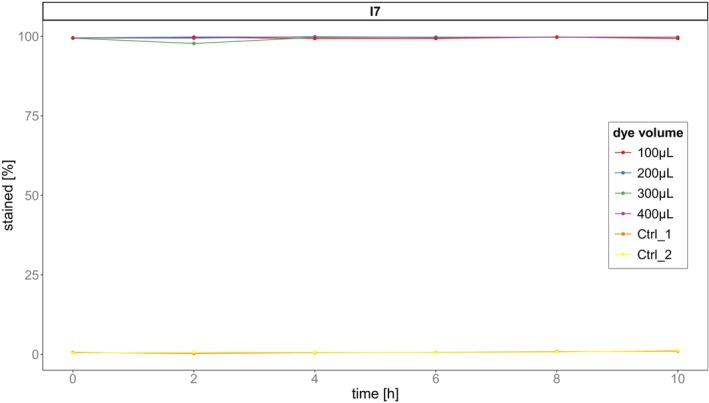
Percentage of stained versus unstained cells at different dye volumes. The relative proportion of stained cells after classification by a fluorescence intensity threshold plotted over time. Cells above the threshold were classified as stained. Colour represents dye volume: Red = 100 μL, blue = 200 μL, Green = 300 μL, Violet = 400 μL, Orange/Yellow = unstained controls.

Over the 10 h of the experiment, the proportion of stained cells classified as stained was high for all dye concentrations (mean = 99.52%, Figure 1, Table S3). Using the 400 μL threshold, the percentage of stained cells was slightly higher than on average (99.628%), and misclassification of unstained cells as stained was low (mean = 0.703%, SE = 0.114). A volume of 400 μL of dye gave the highest *accuracy* and best *average* for the classification and resulted in a consistent classification over time. We therefore continued to use this volume for our competition assay methodology.

### 3.2 Competition Between Genotypes

Based on the established staining procedure we performed a competition assay between two *P. fluorescens* genotypes labelled I6 and I7. Both were isolated from the same population of a previous long‐term evolution experiment (LTEE) and were thus natural competitors. To identify these genotypes on growth agar, they were chosen based on their difference in colony morphology. Genotype I6 produces big, round colonies with fuzzy edges whereas I7 grows small colonies in distorted shapes. For the competition assay, these genotypes were combined at three different initial relative abundances (0.1, 0.5, 0.9) in the absence and presence of the ciliate predator *Tetrahymena thermophila* (each treatment in triplicates).

**FIGURE 2:**
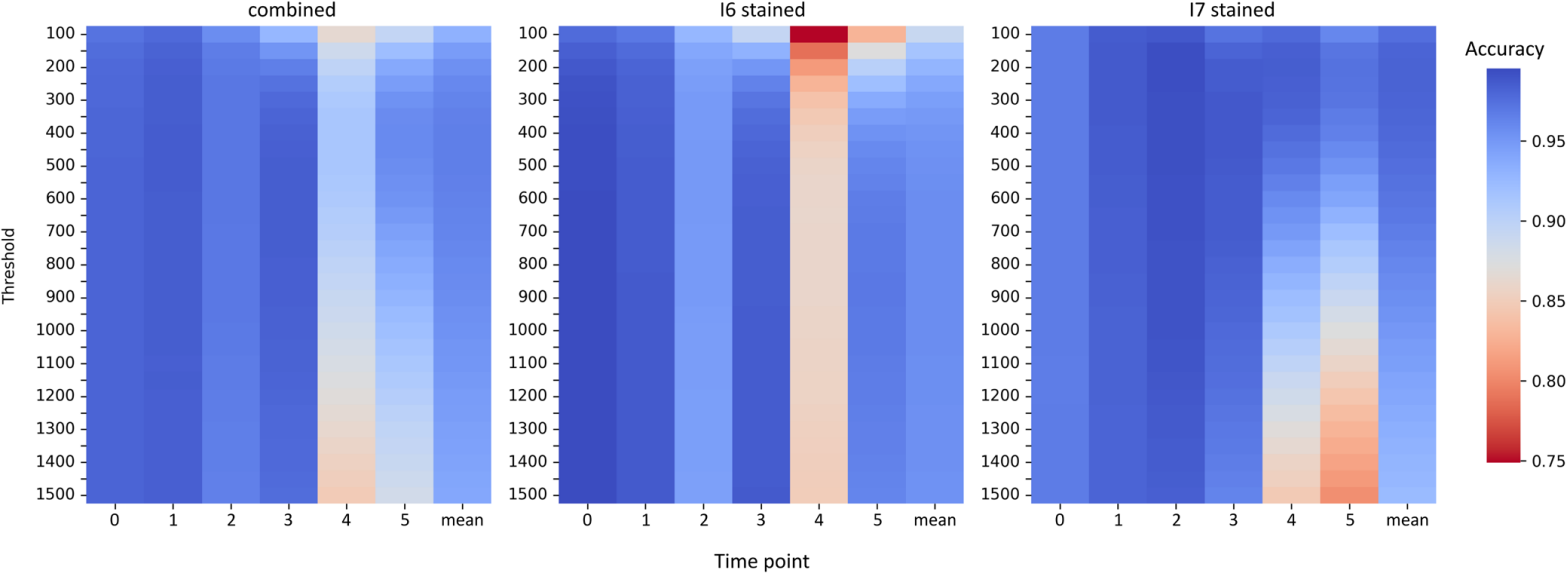
Comparison of classification accuracy between thresholds. For a range of fluorescence intensity thresholds (100–1500) classification accuracy for stained and unstained cells in monocultures is reported in two different scenarios. Combined: Samples with I6 stained and I7 stained are evaluated under the same threshold. I6 stained/I7 stained: Samples with I6 and I7 stained are separately evaluated. Classification accuracy is given for each sampling time point and as mean across all time points.

This competition was followed for 10 h by sampling every 2 h. At each sampling time point, the frequency of the competitors was evaluated based on fluorescence intensity and enumeration of samples plated on growth agar (only 2 of 3 replicates per treatment were plated). For classification of competitors in coculture, we determined separate fluorescence intensity thresholds for each genotype using ROC analysis. For each genotype, all single‐cell fluorescence measurements from a stained monoculture were pooled across time points and compared to the fluorescence of an unstained monoculture of its competitor to construct the ROC curve. The optimal threshold—the intensity maximising overall classification accuracy (mean of true positive rate [TPR] and true negative rate [TNR])—was then applied to coculture data. This yielded thresholds of 858.49 for I6 (TPR = 0.9374, TNR = 0.9808, accuracy = 0.9582) and 201.35 for I7 (TPR = 0.9876, TNR = 0.9767, accuracy = 0.9817). We also tested a single unified threshold for both genotypes, but this reduced overall classification performance (Figure 2), confirming that genotype‐specific thresholds provide the best balance between robustness and accuracy.

### 3.3 Correlation Between Frequencies by Staining and on Growth Agar

**FIGURE 3:**
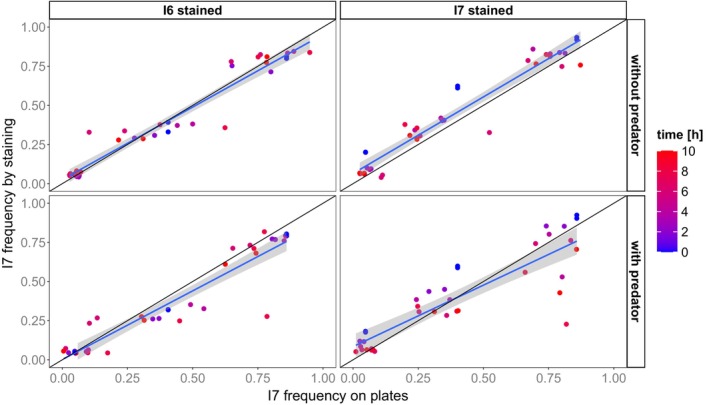
Correlation between genotype frequency on growth agar and classification by fluorescence intensity. Shown is the relative abundance of genotype I7. We used 2 of 3 replicates per treatment (initial abundance, stained genotype, predator presence) for this analysis because the third replicates were not plated. Slopes (blue line) between both estimates were plotted using a linear smooth and derived from coefficients of a linear model: [I6 stained, −Pred] = 0.928; [I6 stained, +Pred] = 0.877; [I7 stained, −Pred] = 0.979; [I7 stained, +Pred] = 0.785 (Table S4). The shaded area illustrates the 95% confidence interval of the slope. The black line illustrates perfect correlation by using a slope of 1.

We evaluated the classification based on fluorescence intensity thresholds by comparing it to the relative abundances of genotypes on growth agar. Both estimates had a highly positive correlation (Pearson coefficient of correlation: *r* = 0.93, *p* < 0.001; Figure 3). Across all time points, the correlation was higher when genotype I6 was stained (*r* = 0.95, *p* < 0.001) than when genotype I7 was stained (*r* = 0.90, *p* < 0.001) but this difference was not significant (*Z* Difference = 1.787, *p* = 0.074). Predator presence significantly lowered the correlation (predator presence: *r* = 0.90, *p* < 0.001; predator absence: *r* = 0.96, *p* < 0.001; *Z* Difference = 2.782, *p* = 0.005).

**FIGURE 4:**
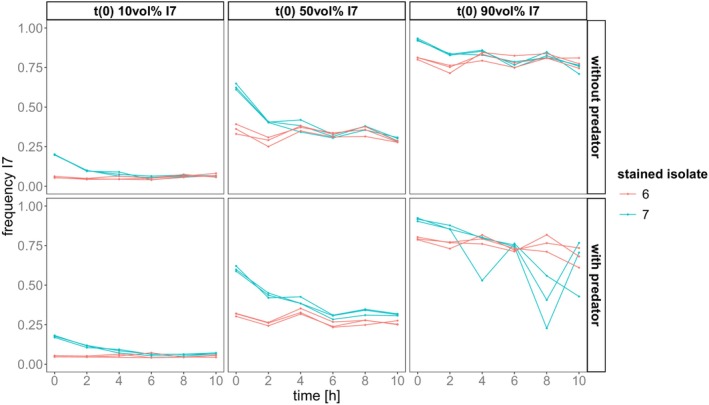
Competition dynamics tracked by classification via fluorescence intensity and shown as relative abundance of genotype I7. Panels show these trajectories dependent on the initial abundance of I7 in vol% of coculture and predator presence/absence. Colour denotes which genotype was stained: Red = I6, Blue = I7.

To evaluate the outcome of competition and its temporal dynamics we analysed relative abundance of genotypes over time based on the fluorescence classification. In the competition assay, the relative abundance of genotype I7 changed significantly over time (Analysis of variance (ANOVA) on Linear mixed effect model (LME), time: *p* < 0.001, *F* = 174.155, DF = 1, Table S5a,b). These changes were influenced by the initial abundance, predator presence/absence and which genotype was stained (interaction *time* with: initial abundance: *p* = 0.001, *F* = 7.874, DF = 2; genotype: *p* < 0.001, *F* = 58.217, DF = 1; predator: *p* = 0.014, *F* = 6.174, DF = 1). Overall, as expected, relative abundances differed between different initial abundances and between the stained genotypes (initial abundance: *p* < 0.001, *F* = 678.932, DF = 2; genotype: *p* < 0.001, *F* = 68.413, DF = 1). In 8 out of 12 combinations (initial abundance, predator, stained genotype), I7 abundance declined significantly (Estimated Marginal Trend (EMT), Figure 4, Table S5c).

**FIGURE 5:**
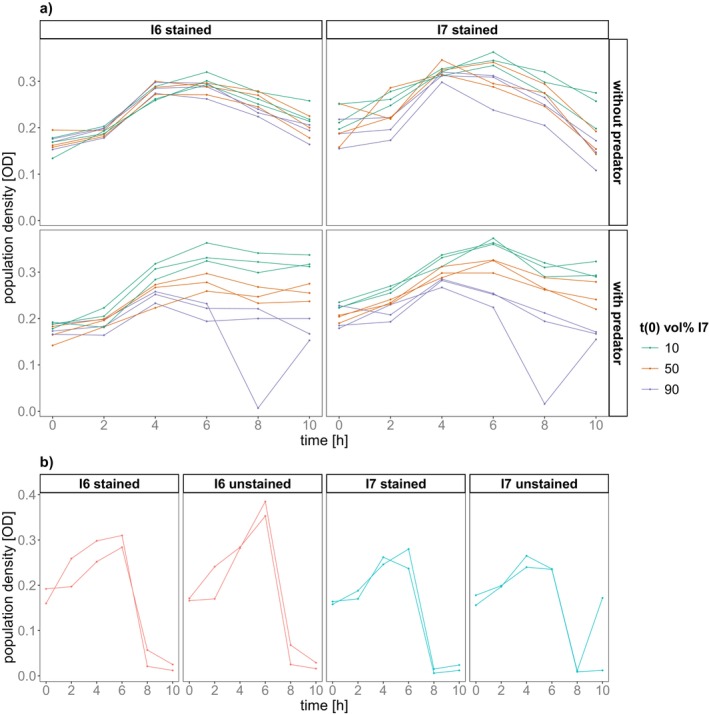
Population dynamics. Population density over time measured by optical density (600 nm) in (a) cocultures and (b) stained/unstained monocultures. For cocultures, colour represents the initial vol% of I7 in culture: Green = 10 vol%, orange = 50 vol%, violet = 90 vol%.

We also estimated population densities in the competition assays through optical density (OD). Differences in population densities can also inform about competition by comparing growth in coculture and monoculture. Bacterial growth began after approx. two hours and the population maximum was reached after two to six hours (Figure 5). Carrying capacity (the maximum OD measured in a sample) differed between different initial abundances and the stained genotype (ANOVA initial abundance: *p* < 0.001, *F* = 53.630, DF = 2; genotype: *p* < 0.001, *F* = 51.746, DF = 1, Table S6a) and was affected by an interaction between predator and initial abundances (*p* < 0.001, *F* = 15.110, DF = 1). Higher initial abundance of I6 led to higher carrying capacity (Estimated Marginal Means, Table S6b), while predator presence amplified the effect of initial abundances on carrying capacity and led to more diverging densities (Figure 5).

### 3.4 Competition Between Species

**FIGURE 6:**
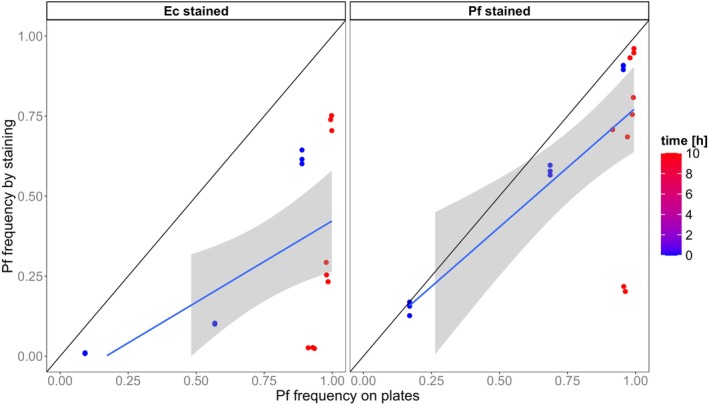
Correlation between species frequency on growth agar and classification by fluorescence intensity. Shown is the relative abundance of *Pseudomonas fluorescence*. Slopes (blue line) between both estimates were plotted using a linear smooth and derived from coefficients of a linear model: Ec stained = 0.509; Pf stained = 0.744 (Table S7). Abbreviations: Ec = *E.coli*, Pf = *P.fluorescens*. The shaded area illustrates the 95% confidence interval of the slope. The black line illustrates perfect correlation by using a slope of 1.

We next applied our method to test competition between two bacterial species. We chose clonal strains of *E. coli* and *P. fluorescens* because both species differ in colony morphology on our growth agar. Again, stained and unstained competitors were combined at three different initial relative abundances (0.1, 0.5, 0.9). Their competition was then followed over 10 h and sampled every 2 h to measure fluorescence intensity. Start and endpoint samples were plated on growth agar. For classification of competitors in coculture, we determined a fluorescence threshold for each species based on all time points by comparing a stained monoculture to an unstained monoculture of its competitor (Ec stained: threshold = 320.42, TPR = 0.9982, TNR = 0.9938, accuracy = 0.9964; Pf stained: threshold = 378.18, TPR = 0.9977, TNR = 0.9879, accuracy = 0.9927). Comparing fluorescence‐based classification to the one on growth agar, we found positive correlation (*r* = 0.6, *p* < 0.001, Figure 6). The correlation was stronger when *P. fluorescens* was stained (*r* = 0.73, *p* < 0.001) than when *E. coli* was stained (*r* = 0.58, *p* = 0.012).

**FIGURE 7:**
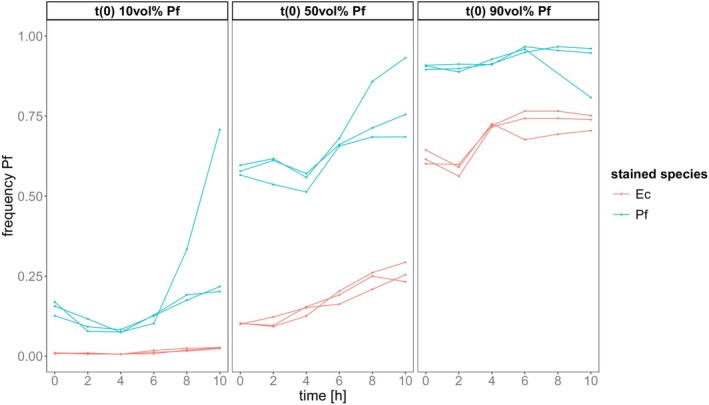
Competition dynamics tracked by classification via fluorescence intensity. Relative abundance of *Pseudomonas fluorescens* is plotted over time. Panels show these trajectories dependent on the initial abundance of *Pseudomonas fluorescens* in vol% of co‐culture. Colour denotes which species was stained: Red = Ec, Blue = Pf.

As for the genotype competition, we analysed the relative abundance of species over time based on the fluorescence classification. The relative abundance of *P. fluorescens* changed significantly over time (ANOVA on LME, time: *p* < 0.001, *F* = 52.424, DF = 1, Table 8ab). These dynamics differed across the initial abundance of *P. fluorescens* (interaction *time* with: initial abundance: *p* = 0.038, *F* = 3.409, DF = 2). Overall, the species' relative abundance differed between initial abundances and between the stained species (initial abundance: *p* < 0.001, *F* = 93.979, DF = 2; species: *p* < 0.001, *F* = 37.465, DF = 1). In 5 out of 6 treatment combinations, *P. fluorescens* abundance increased significantly (Figure 7, Table S8c).

**FIGURE 8:**
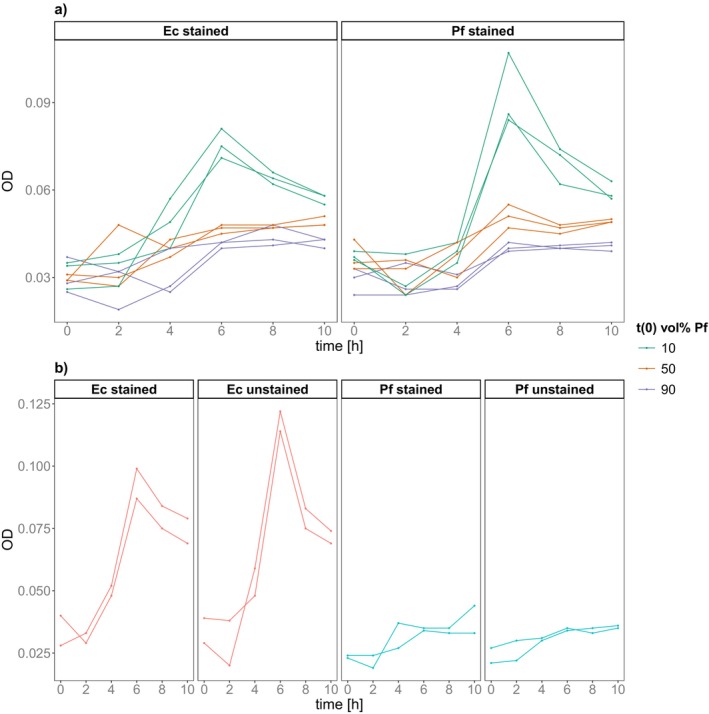
Population dynamics. Population density over time measured by optical density (600 nm) in (a) competition co‐cultures and (b) stained/unstained mono‐cultures. For co‐cultures colour represents the initial vol% of *Pseudomonas fluorescens* in culture: Green = 10 vol%, Orange = 50 vol%, Violet = 90 vol%. Abbreviations: Ec = *Escherichia coli* , Pf = *Pseudomonas fluorescens* .

We estimated population densities in competition by measuring their optical density (OD). Bacterial growth in all cultures started after two hours and had a maximum between two and six hours (Figure 7). Carrying capacity (max OD) differed between different species and initial abundances but was not dependent on the species stained (ANOVA, initial abundance: *p* < 0.001, *F* = 82.079, DF = 2; species: *p* = 0.08, *F* = 3.663, Table S9a), although there was a significant interaction between both (*p* = 0.034, *F* = 4.521, DF = 2). Overall, carrying capacity was higher with initially higher abundance of *Escherichia coli* (Figure 8, Estimated Marginal Means, Table S9b).

## 4 Discussion

We present a novel, high‐throughput method for tracking bacterial competitors in pairwise competition using a transient fluorescent dye. Our results show that stained and unstained cells can be reliably and objectively classified for at least four generations, allowing the tracking of competing genotypes and species in coculture. Using this method, we detected frequency changes between competitors and demonstrated that the outcome of competition was influenced by initial relative abundances, and for competing genotypes, also by predator presence. Overall, our method effectively quantifies pairwise bacterial competition and provides sufficient sensitivity to resolve frequency‐dependent fitness effects across generations.

To classify stained and unstained cells, we applied a ROC‐based approach to derive data‐driven intensity thresholds. The ROC threshold provides an objective criterion for separating stained and unstained populations, replacing fixed or visually defined cutoffs typically used in fluorescence‐based methods. This approach maximises classification accuracy given the measured overlap between distributions while maintaining high reproducibility across experiments. ROC thresholds were initially derived from monocultures where class membership is known and then applied to cocultures. Because class identity in mixed samples is unknown, ROC analysis cannot be directly applied during coculture without additional labelling. To ensure threshold robustness, we compared fluorescence retention between monocultures and cocultures and found no substantial difference in the temporal decay of mean fluorescence per stained cell (Figures S4–S7). This indicates that coculture conditions do not alter fluorescence dynamics, supporting the stability of monoculture‐derived thresholds across experiments.

We further evaluated the trade‐off between threshold complexity and analytical efficiency. Applying separate monoculture‐derived thresholds for each stained competitor significantly improved accuracy without added effort (Figure 2). In contrast, recalculating thresholds for each time point yielded only marginal improvement (see Figures S8–S11 for comparisons of ROCs at different time points), as the global threshold – derived from pooled data over time – already captured the gradual signal decline. This design thus balances classification reliability with analytical simplicity, ensuring consistent discrimination of stained and unstained cells throughout the experiment.

To test for potential consequences of physiological effects of the dye, we compared growth parameters between stained and unstained monocultures that ran in parallel with competition assays. These comparisons revealed only minor differences between treatments (Tables S10, S11), indicating that the staining procedure exerted no substantial or measurable impact on cellular performance under our experimental conditions. At the same time, it is important to consider whether cell physiology could, in turn, influence fluorescence performance. While individual cells may experience minor fluorescence intensity changes due to membrane turnover, fluorophore degradation, or photobleaching, these effects remain overall small in magnitude when compared to the predictable halving of fluorescence signal that occurs with each cell division. Consequently, such physiological effects do not meaningfully compromise the classification accuracy. Nonetheless, minor temporal variations in fluorescence classification could still emerge from other sources, including background fluorescence in the culture medium, biofilm formation, presence of metabolites or molecules specifically present in cocultures and inaccuracies in measurement and detection.

Validation against plating‐based reference assays revealed strong correlations between fluorescence‐derived and colony‐count–derived frequencies for genotypic competitions but weaker correlations for *P. fluorescens–E. coli* competitions. The lower agreement in interspecific assays may stem from differential staining efficiency between species. However, time‐resolved threshold analyses showed stable intensity distributions and no sign of dye fading, suggesting that the difference may arise from biases in the plate‐based reference. Because *P. fluorescens* formed more colonies than *E. coli* on solid media, plate‐derived frequencies may have underestimated *E. coli* abundance. Importantly, this bias was not driven by use of a different medium for the plating‐based reference than for the competition (see supplementary results). Instead, growth differences between liquid and solid media (Cuny et al. 2007; Fortuin et al. 2020; Partridge et al. 2019) or variations in surface adhesion due to cell wall composition (Bakholdina et al. 2001; Giwercman et al. 1992; Walsh and Moran 1997) could also explain this discrepancy. Future work could test this by growing stained monocultures under addition of coculture filtrates or by using an independent sequencing‐based reference (e.g., 16S rRNA amplicon sequencing; Howells et al. 2025).

Small deviations between replicate competitions may also stem from slight density differences in inoculations of stained and unstained cultures, despite both being derived from the same source. Such variations can occur during washing steps and splitting of cultures prior to competition and likely explain the small but consistent frequency discrepancies observed at early time points.

While our approach performs robustly across experimental contexts, it also has practical limitations that should be noted. The transient nature of the dye restricts reliable classification to short‐ to mid‐term assays (approximately up to four to five generations) because fluorescence intensity necessarily declines with cell division. This limits applicability in long‐term or stationary‐phase competitions. The method also currently resolves only two competitors at a time, as overlapping fluorescence spectra would hinder multi‐species discrimination. Furthermore, although ROC thresholds derived from monocultures remain stable under our conditions, more complex community settings might require additional calibration or adaptive thresholding. Addressing these aspects in future work could further extend the method's scope and precision.

Quantifying competition outcomes among bacteria remains a central methodological challenge. In recent years, research has revealed increasing complexity in the ecological and evolutionary processes shaping microbial communities (De Meester et al. 2019; Hoffmann et al. 2025; Kivikoski et al. 2025; Røder et al. 2020). Numerous abiotic and biotic factors influence inter‐ and intraspecific competition (Nadell et al. 2016; Widder et al. 2016). Pairwise competition assays continue to play a key role in understanding these processes. While predicting community dynamics from pairwise interactions alone can be difficult (Levine et al. 2017; McClean et al. 2019), such experiments remain essential for linking genotypes, traits and ecological outcomes (Carrara et al. 2015; Friedman et al. 2017; Good et al. 2017; Lang et al. 2013; Maharjan et al. 2015; Kičiatovas et al. 2026).

In addition to ecological applications, this method is particularly valuable in experimental evolution research, where newly evolved isolates often differ only subtly in genotype or phenotype and may lack selectable or fluorescent markers (Friman et al. 2008; Meroz et al. 2024). Because our approach requires no genetic modification or prior genomic information, it allows direct competition tests among evolved clones or populations as they emerge, providing a fast and unbiased way to quantify realised fitness differences (see Table S12 for a comparison with genetic labelling and sequencing‐based approaches). This simplifies post‐evolution phenotyping and enables systematic testing of adaptive dynamics across multiple evolved lines.

Beyond its immediate application to transient fluorescence assays, the ROC‐based classification framework we present provides a generalisable approach for quantitative threshold definition in single‐cell datasets. It could be extended to other fluorescence‐based measurements – such as metabolic reporters, viability stains, or stress indicators – offering a standardised, data‐driven way to distinguish overlapping cellular states. Together, these features make transient dye staining coupled with ROC analysis a flexible, scalable and broadly applicable framework for high‐throughput quantification of microbial competition and related ecological or evolutionary processes.

## Author Contributions

**Julius Hoffmann:** conceptualization (equal), formal analysis (equal), investigation (lead), methodology (lead), software (equal), visualization (equal), writing – original draft (lead), writing – review and editing (equal). **Ole Johannsen:** formal analysis (equal), methodology (equal), software (equal), visualization (equal). **Lutz Becks:** conceptualization (equal), formal analysis (equal), funding acquisition (lead), methodology (equal), resources (lead), supervision (lead), visualization (equal), writing – original draft (supporting), writing – review and editing (equal).

## Funding

This work was supported by Deutsche Forschungsgemeinschaft, BE 4135/12‐1.

## Disclosure

Statement on inclusion: Our manuscript presents a lab‐based study without link to a geographic region or locality. All authors are (or were) residents of the city/region where the experiments were performed.

## Conflicts of Interest

The authors declare no conflicts of interest.

## Supporting information


**Figure S1:** Fluorescence staining: ROC all time points & all dye volumes.
**Figure S2:** Fluorescence staining: ROC TP0 for all dye volumes.
**Figure S3:** Fluorescence staining: ROC TP5 for all dye volumes.
**Figure S4:** Genotype competition–mean fluorescence intensity in monocultures.
**Figure S5:** Species competition–mean fluorescence intensity in monocultures.
**Figure S6:** Genotype competition–retention curves in mono‐ and cocultures.
**Figure S7:** Species competition–retention curves in mono‐ and cocultures.
**Figure S8:** Species competition: ROC *P. fluorescence* TP0‐TP5.
**Figure S9:** Species competition: ROC *E. coli* TP0‐TP5.
**Figure S10:** Genotype competition: ROC I7 TP0‐TP5.
**Figure S11:** Genotype competition: ROC I6 TP0‐TP5.
**Table S1:** Fluorescence staining: threshold performance single TP vs. general.
**Table S2:** Fluorescence staining: threshold performance dye volumes.
**Table S3:** Fluorescence staining: proportion of stained cells depending on dye volume.
**Table S4:** Genotype competition: LM classification fluorescence vs. plate.
**Table S5:** (a–c) Genotype competition: LME genotype frequency.
**Table S6:** (a, b) Genotype competition: LM carrying capacity.
**Table S7:** Species competition: LM classification fluorescence vs. plate.
**Table S8:** (a–c) Species competition: LME species frequency.
**Table S9:** (a, b) Species competition: LM carrying capacity.
Supplementary Results & Analyses.

## Data Availability

All data and code for analyses are available on Zenodo: https://doi.org/10.5281/zenodo.18822613 (Version 2).
