# Supplementary material for: Tracking Inter‐ and Intraspecific Bacterial Competition in Pairwise Assays Using a Transient Fluorescent Dye
Source: Ecol Evol. 2026 Apr 7;16(4):e73395. doi: 10.1002/ece3.73395 (PMC13058240; doi:10.1002/ece3.73395)
Supplement: Supplementary file 1 — Figure S1: Fluorescence staining: ROC all time points & all dye volumes. Figure S2: Fluorescence staining: ROC TP0 for all dye volumes. Figure S3: Fluorescence staining: ROC TP5 for all dye volumes. Figure S4: Genotype competition–mean fluorescence intensity in monocultures. Figure S5: Species competition–mean fluorescence intensity in monocultures. Figure S6: Genotype competition–retention curves in mono‐ and cocultures. Figure S7: Species competition–retention curves in mono‐ and cocultures. Figure S8: Species competition: ROC P. fluorescence TP0‐TP5. Figure S9: Species competition: ROC E. coli TP0‐TP5. Figure S10: Genotype competition: ROC I7 TP0‐TP5. Figure S11: Genotype competition: ROC I6 TP0‐TP5. Table S1: Fluorescence staining: threshold performance single TP vs. general. Table S2: Fluorescence staining: threshold performance dye volumes. Table S3: Fluorescence staining: proportion of stained cells depending on dye volume. Table S4: Genotype competition: LM classification fluorescence vs. plate. Table S5: (a–c) Genotype competition: LME genotype frequency. Table S6: (a, b) Genotype competition: LM carrying capacity. Table S7: Species competition: LM classification fluorescence vs. plate. Table S8: (a–c) Species competition: LME species frequency. Table S9: (a, b) Species competition: LM carrying capacity. Supplementary Results & Analyses. [file ECE3-16-e73395-s001.docx]

Supplementary Information

for

Tracking inter- and intraspecific bacterial competition in pairwise assays using a transient fluorescent dye

**This file includes (in this order):**

Supplementary Results & Analyses

Supplementary Figures

Figure S1 – Fluorescence staining: ROC all time points & all dye volumes

Figure S2 – Fluorescence staining: ROC TP0 for all dye volumes

Figure S3 – Fluorescence staining: ROC TP5 for all dye volumes

Figure S4 – Genotype competition - mean fluorescence intensity in monocultures

Figure S5 – Species competition - mean fluorescence intensity in monocultures

Figure S6 – Genotype competition - retention curves in mono- and cocultures

Figure S7 – Species competition - retention curves in mono- and cocultures

Figure S8 – Species competition: ROC *P. fluorescence* TP0-TP5

Figure S9 – Species competition: ROC *E. coli* TP0-TP5

Figure S10 – Genotype competition: ROC I7 TP0-TP5

Figure S11 – Genotype competition: ROC I6 TP0-TP5

Supplementary Tables

Table S1 - Fluorescence staining: threshold performance single TP vs general

Table S2 - Fluorescence staining: threshold performance dye volumes

Table S3 - Fluorescence staining: proportion of stained cells depending on dye volume

Table S4 - Genotype competition: LM classification fluorescence vs plate

Table S5a,b,c - Genotype competition: LME genotype frequency

Table S6a,b - Genotype competition: LM carrying capacity

Table S7 - Species competition: LM classification fluorescence vs plate

Table S8a,b,c - Species competition: LME species frequency

Table S9a,b - Species competition: LM carrying capacity

Supplementary Results & Analyses

Plating-bias control experiment

Having observed a discrepancy between staining-based and plating-based frequency estimates for the species competition we performed a control experiment to assess the possible bias of plate-based fitness estimates introduced by using alternate media for our competition experiments in liquid culture (5% KB medium) and our plating-based reference (50% PPY medium). We aimed to detect differential growth of *E.coli* and *P. fluorescens* on growth agar (evaluated as the number of growing colonies) prepared with 5% KB medium or 50% PPY medium. To align with the competition experiment we revived both species from our glycerol stocks and preconditioned them in 5% King’s B (KB) medium overnight. Then we plated dilution series of the cultures (1:10, 1:100,1:1000, 1:10 000, 1:100 000, 1:1 000 000) on growth agar prepared with either media (three replicates each) and counted the number of colonies after 24h of growth (KB plates after 48h) at 28°C. Generally, both species grew better on PPY agar. There were 2.37 times more *E. coli* colonies on PPY growth agar (mean=452.33, SD=13.61) than on KB agar (mean=190.67, SD=56.36) and 1.55 times more *P. fluorescence* (mean=380.33, SD=140.22) colonies on PPY growth agar than on KB growth agar (mean=246, SD= 8.16). Comparing the abundance of *E. coli* and *P. fluorescens* colonies on the different media using one-sided T-tests, we found a significant effect for *E. coli* (p=0.001) but not *P.fluorescens* (p=0.086). This suggests that for these species there might be a slight bias introduced by choosing a different medium for our plate-based reference. However, in our experiment we observed more *P. fluorescence* on plates than estimated by our staining-based approach. The observed bias would rather work against this overrepresentation. Thus, this bias is an unlikely contributor to the discrepancy observed between staining- and plating-based estimates.

Growth curve parameters from stained and unstained monocultures

To evaluate potential physiological effects of staining with the fluorescent dye, we estimated growth parameters for both genotypes and species under stained and unstained conditions. For each genotype/species and staining condition, two replicate monocultures were monitored in parallel with the respective competition experiments. Polynomial growth models (*function ipg_multisample*, R package *ipolygrowth*) were fitted to the recorded growth curves (Figures 5b & 8b), and growth parameters were extracted from these fits (Tables S10, S11). Differences in growth rate between stained and unstained cultures were assessed by bootstrapping the fitted curves. For each bootstrap sample, we calculated the difference in estimated growth rate (stained – unstained) and considered it significant when the 95% confidence interval of this distribution excluded zero (equivalent to p < 0.05). Given that only two biological replicates per condition were available, this analysis primarily reflects model-based uncertainty rather than full biological variance, and results should be interpreted as indicative.

The resulting 95% confidence intervals for the growth rate difference (stained –  unstained) were:  I6 = [–0.0477, –0.0103];  I7 = [–0.0005, 0.0192];  *E. coli*= [–0.0156, –0.0031];  *P. fluorescens*= [–0.0011, 0.0056]. A significant difference between stained and unstained growth rates was detected for genotype I6 and *E. coli*, where the negative confidence intervals indicate slightly slower growth of stained cells. For genotype I7 and *P.  fluorescens*, confidence intervals included zero, suggesting no measurable effect of staining on growth rate under the tested conditions. The small but statistically detectable reductions in growth rate were found for genotype I6 and E. coli when stained, suggesting a minor physiological cost of dye exposure. However, the magnitude of these effects was low and did not affect classification accuracy or relative fitness outcomes during competition, indicating that staining had negligible impact on the assay’s overall validity.

Supplementary Figures

**Figure S1. ROCs for thresholds based on all time points at all dye volumes.** Based on all time points a fluorescence threshold at each dye volume was determined to classify stained and unstained cells of genotype I7 (each in monocultures). The optimal threshold for each dye volume is marked by a black dot on the corresponding ROC curve. Colours represent dye volumes for which the ROC was drawn. With 400µl dye the threshold is highest, which suggests that stained cells had higher fluorescence compared to the other dye volumes.
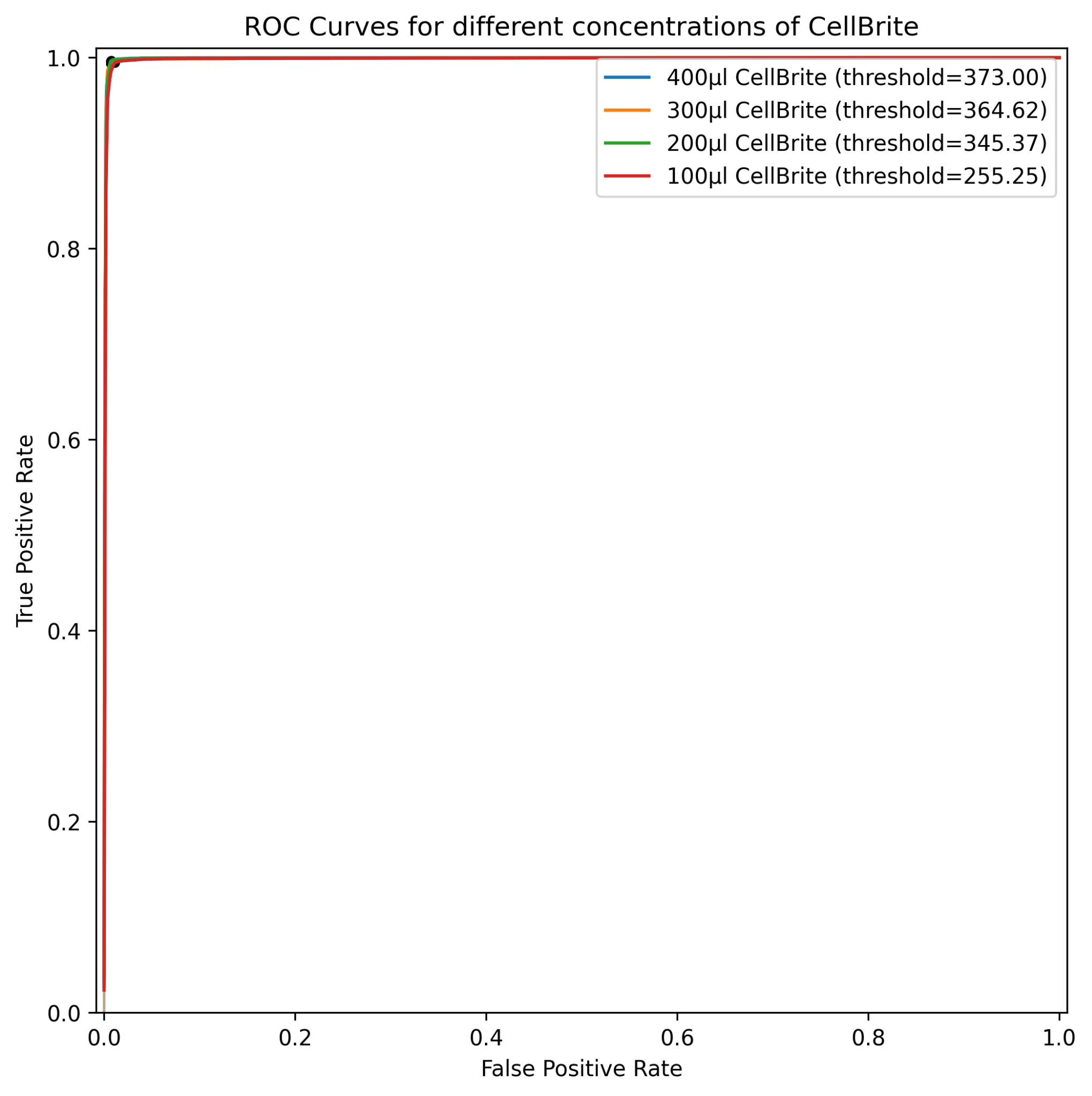


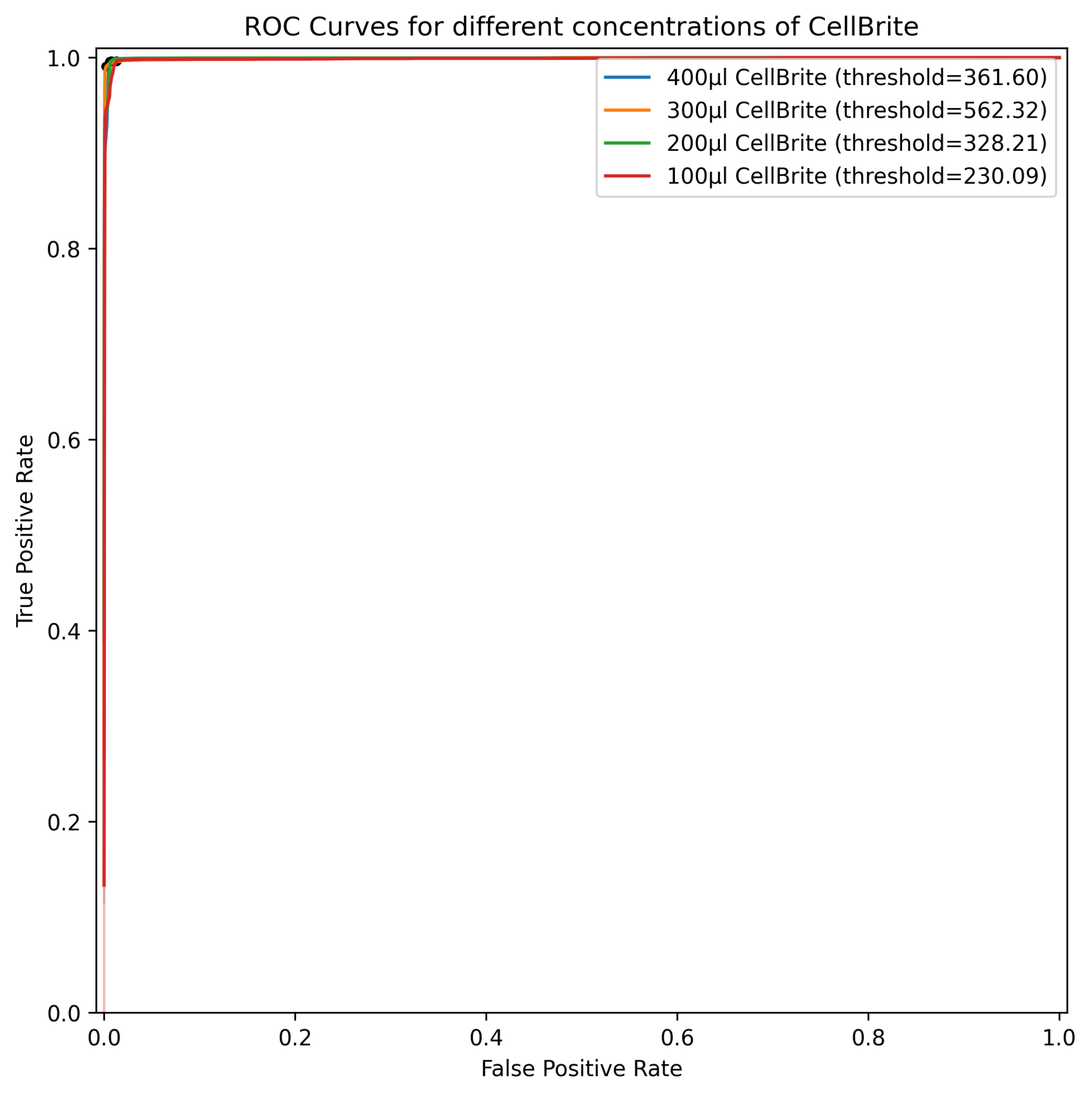


**Figure S2. ROCs for thresholds based on time point TP0 at all dye volumes.** Based on TP0 a fluorescence threshold at each dye volume was determined to classify stained and unstained cells of genotype I7 (each in monocultures). The optimal threshold for each dye volume is marked by a black dot on the corresponding ROC curve. Colours represent dye volumes for which the ROC was drawn.

**Figure S3. ROCs for thresholds based on time point TP5 at all dye volumes.** Based on TP5 a fluorescence threshold at each dye volume was determined to classify stained and unstained cells of genotype I7 (each in monocultures). The optimal threshold for each dye volume is marked by a black dot on the corresponding ROC curve. Colours represent dye volumes for which the ROC was drawn.
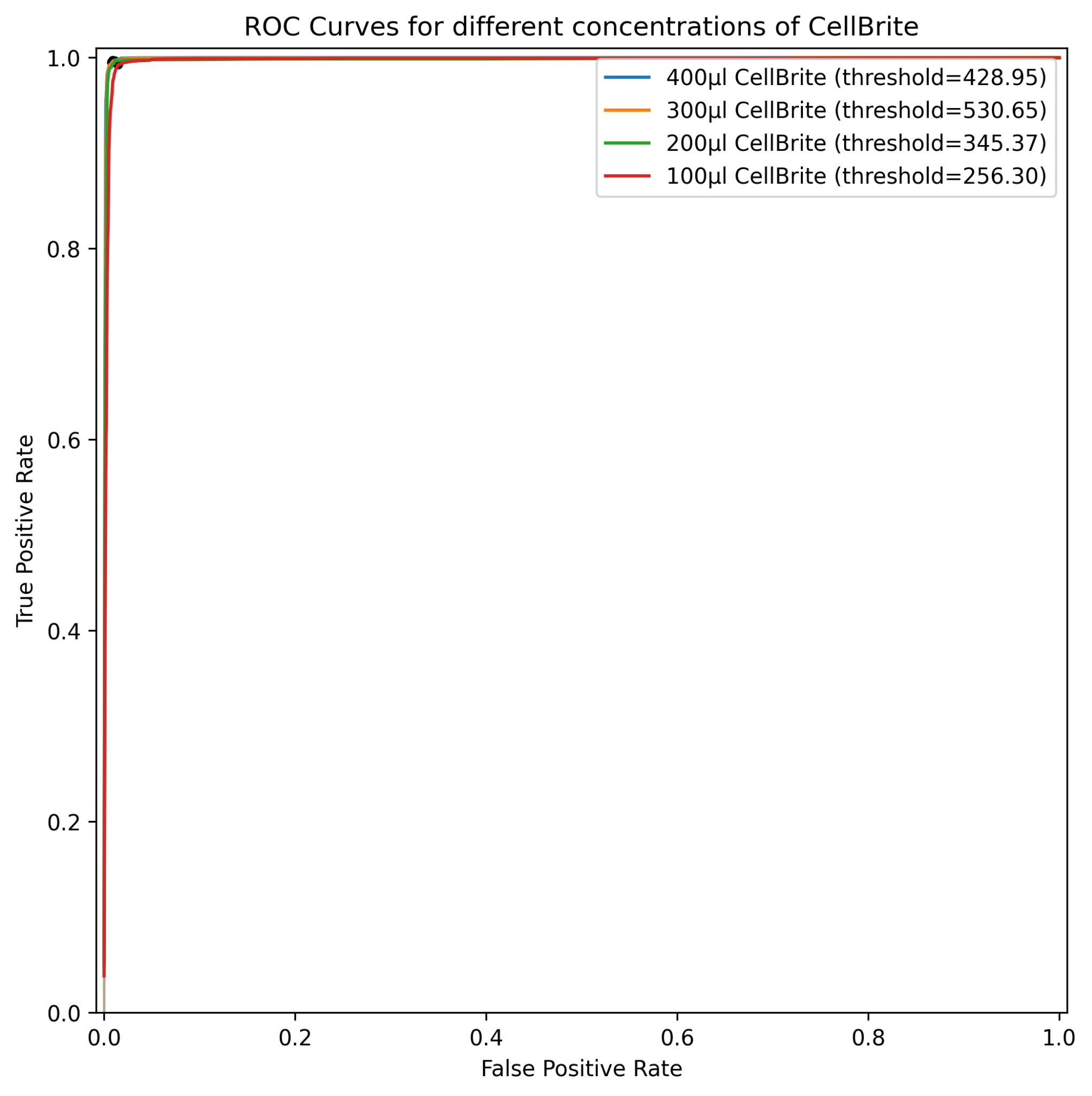


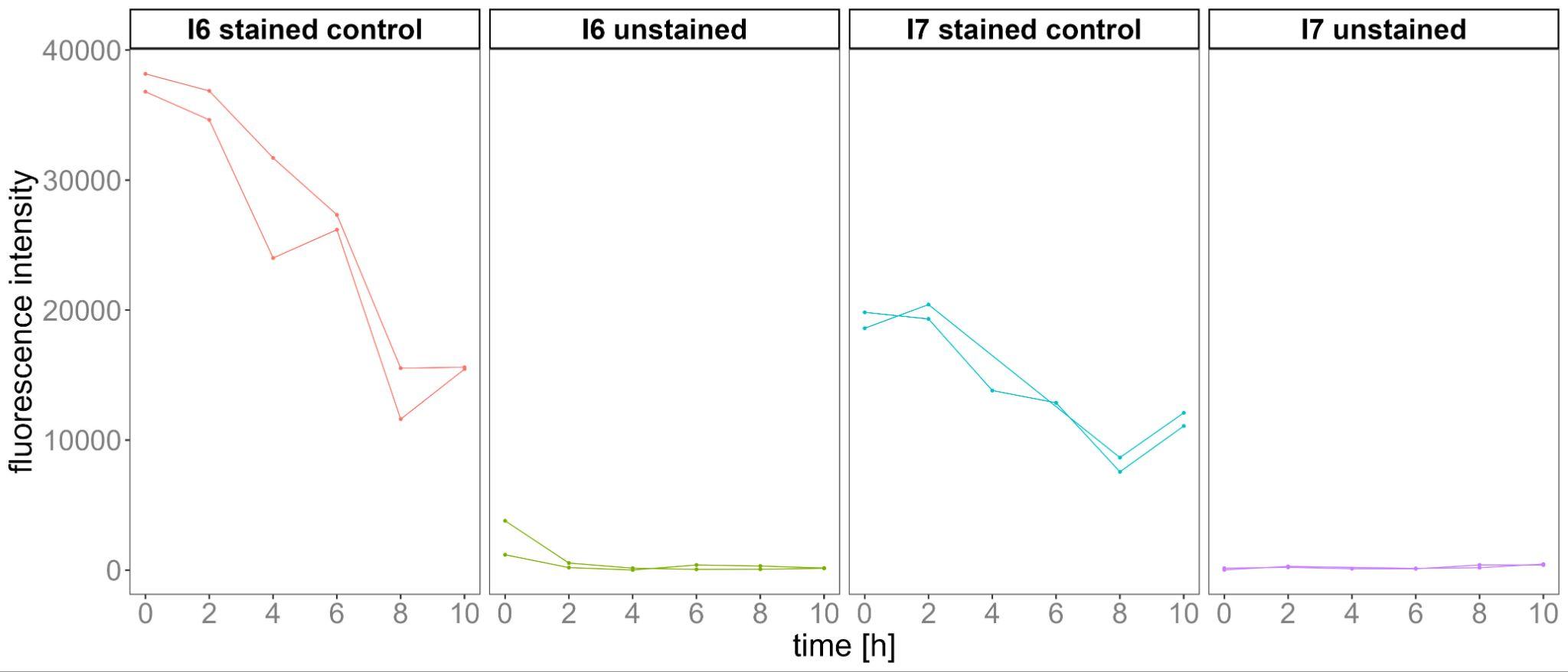


**Figure S4. Genotype competition - mean fluorescence intensity in monocultures.** Mean fluorescence intensity per cell over time is shown for either stained or unstained monocultures of the genotypes I6 and I7. Colours denote staining condition and genotype: red=I6 stained, green=I6 unstained, blue=I7 stained, purple=I7 unstained. For each case two technical replicates are displayed.


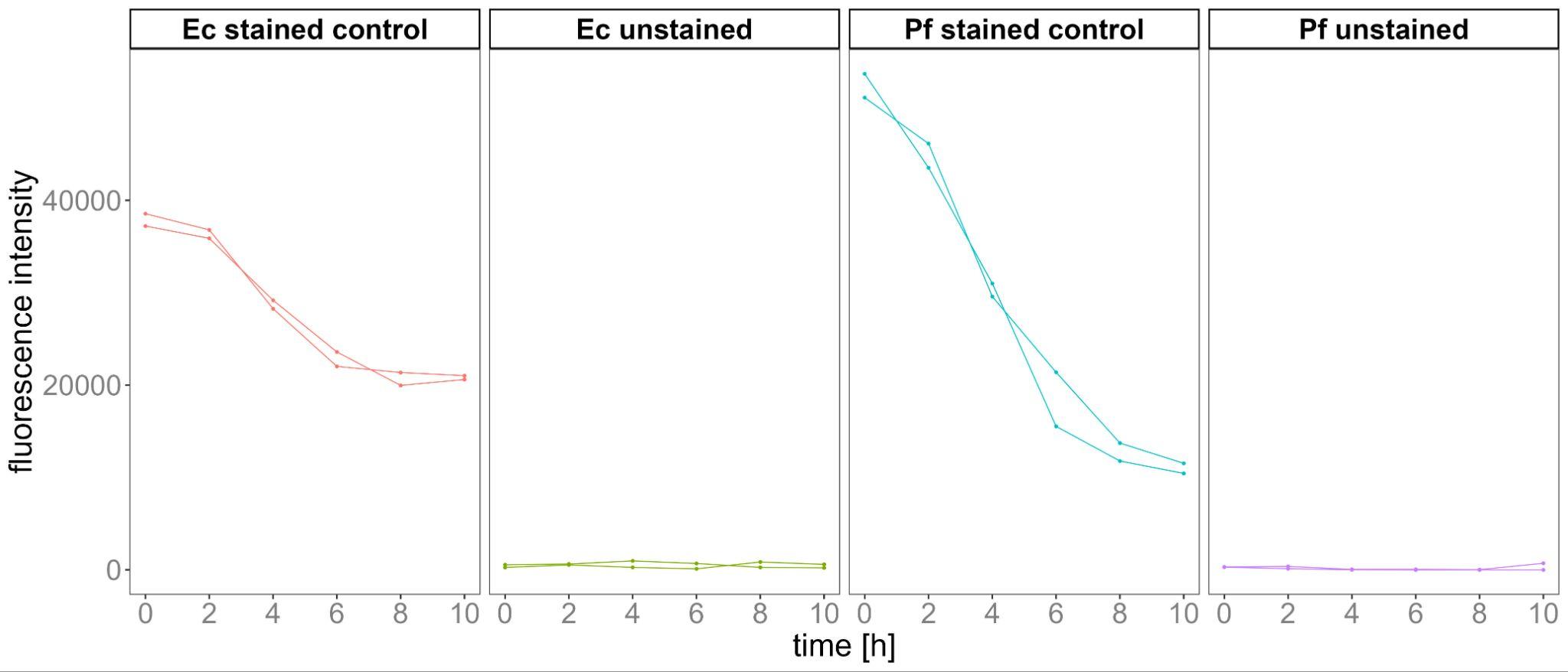


**Figure S5. Species competition - mean fluorescence intensity in monocultures.** Mean fluorescence intensity per cell over time is shown for either stained or unstained monocultures of the two species *E.coli* and *P.fluorescens*. Colours denote staining condition and genotype: red=*E.coli* stained, green=*E.coli* unstained, blue=*P.fluorescens* stained, purple=*P.fluorescens* unstained. For each case two technical replicates are displayed.


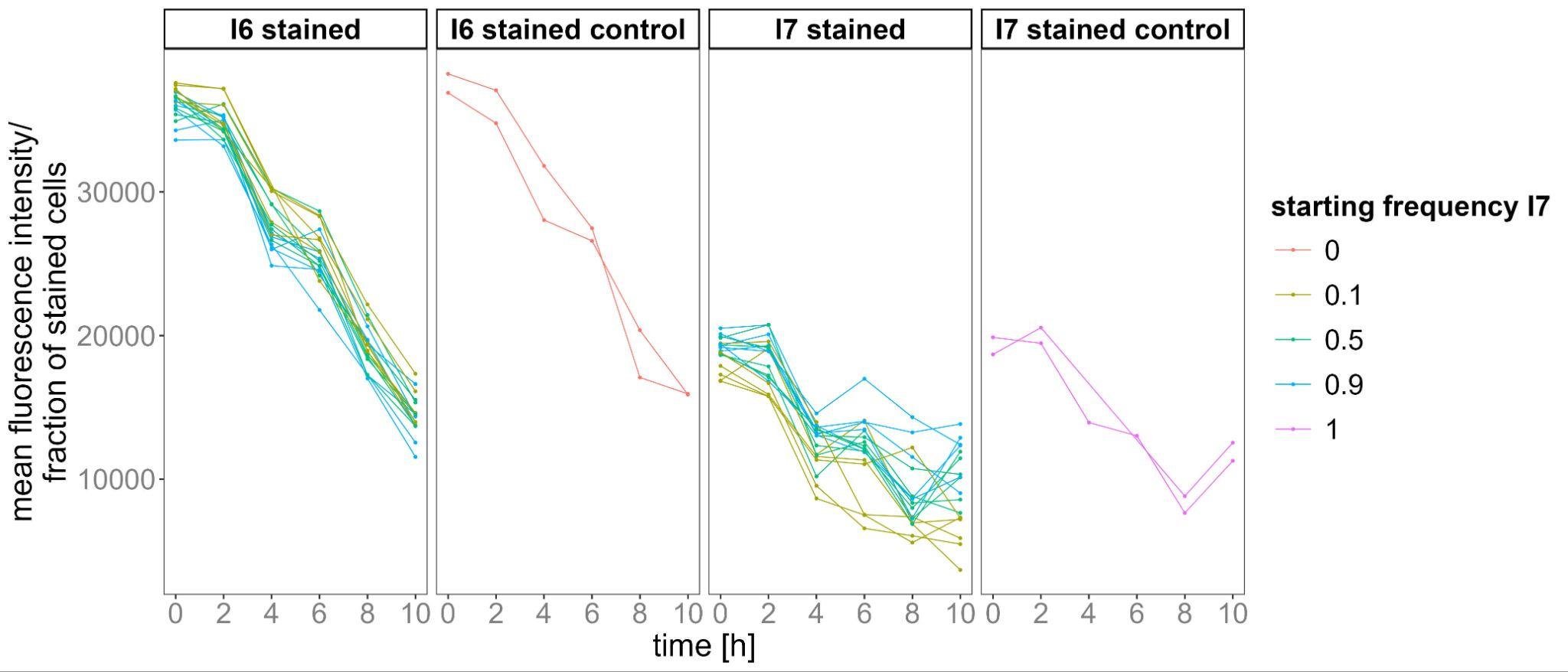


**Figure S6.** **Genotype competition - fluorescence retention curves in mono- and cocultures.** Shown is the retention of fluorescence signal in stained cells over time expressed as the mean fluorescence intensity in a given culture divided by the fraction of stained cells (derived from our thresholding approach). This way fluorescence retention is comparable between mono- and cocultures. Data from monocultures is derived from each two stained monoculture controls per genotype during the competition experiment. Coculture data is derived from the competition cultures of the experiment. Colours denote the different experimental treatments described by the theoretical initial frequency of genotype I7 in a given culture: red=0, olive=0.1, green=0.5, blue=0.9, pink=1.


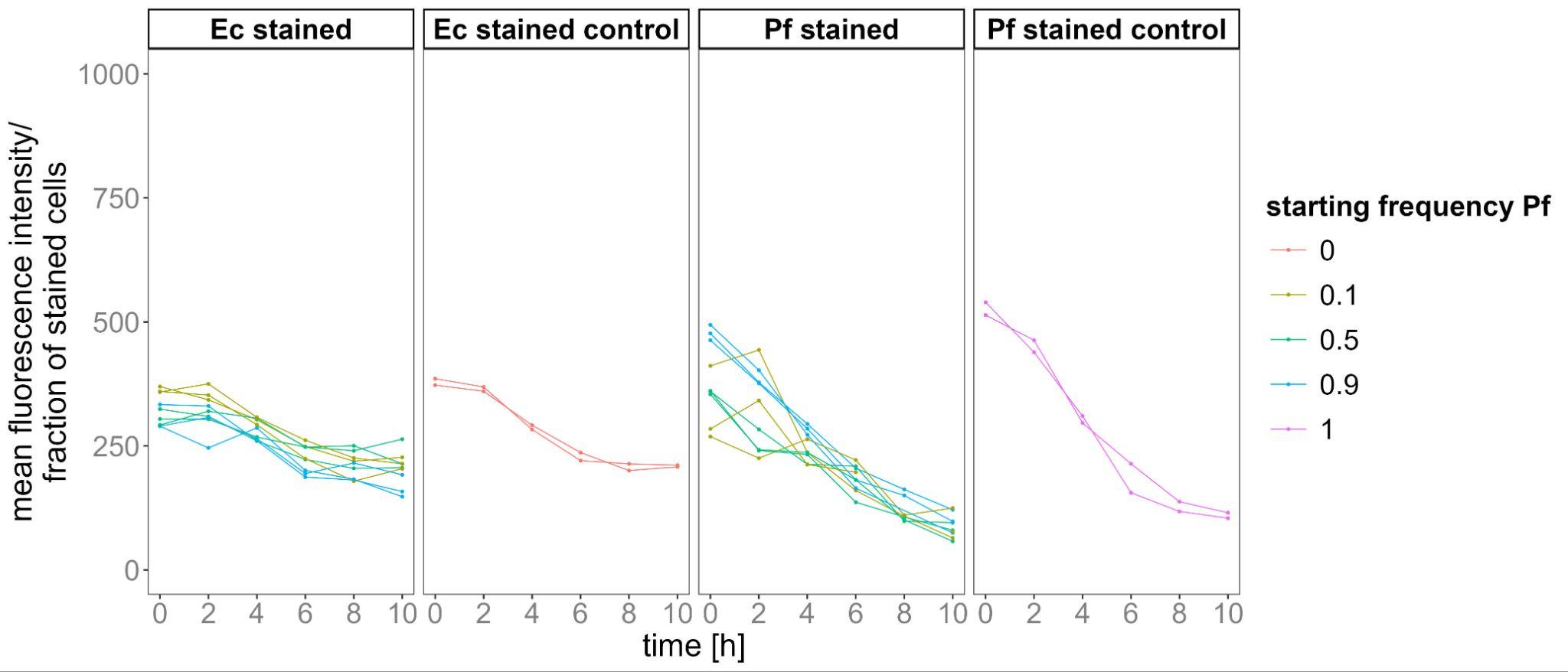


**Figure S7. Species competition - fluorescence retention curves in mono- and cocultures.** Shown is the retention of fluorescence signal in stained cells over time expressed as the mean fluorescence intensity in a given culture divided by the fraction of stained cells (derived from our thresholding approach). This way fluorescence retention is comparable between mono- and cocultures. Data from monocultures is derived from each two stained monoculture controls per species during the competition experiment. Coculture data is derived from the competition cultures of the experiment. Colours denote the different experimental treatments described by the theoretical initial frequency of *P.fluorescens* in a given culture: red=0, olive=0.1, green=0.5, blue=0.9, pink=1.

**Figure S8. Species competition - ROC curves for *Pseudomonas fluorescens* stained on time points TP0-TP5.** Based on a ROC curve at each time point a separate threshold was determined for the classification between stained Pf and unstained Ec (monocultures). The optimal threshold for each time point (black dots) is plotted next to the general threshold (black cross) on each ROC curve. Colours represent time points for which the ROC was drawn. The general threshold is very close to the optimal threshold at each time point. Note: x- and y-axis are zoomed in to resolve minute differences in ROC curves.
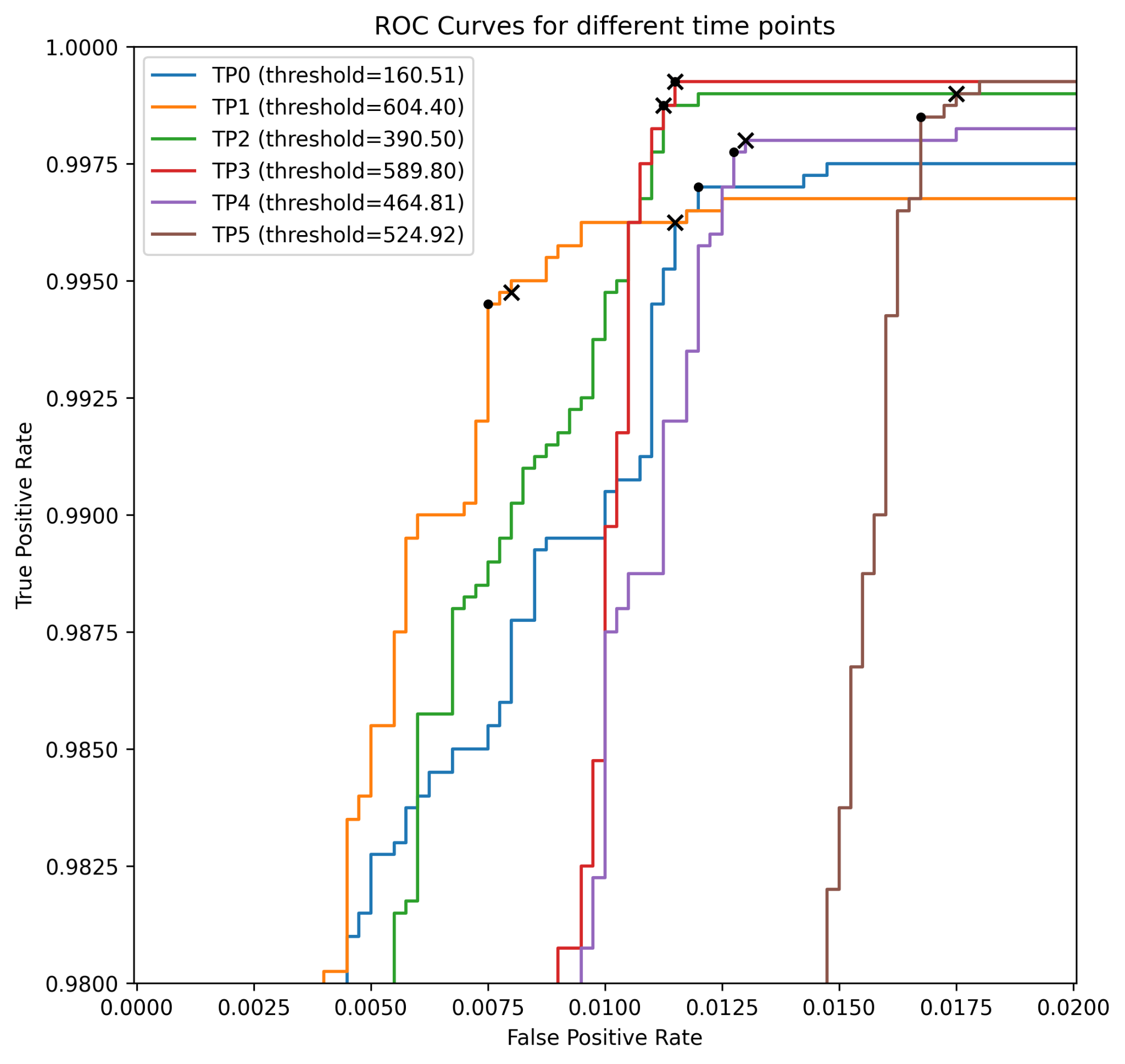


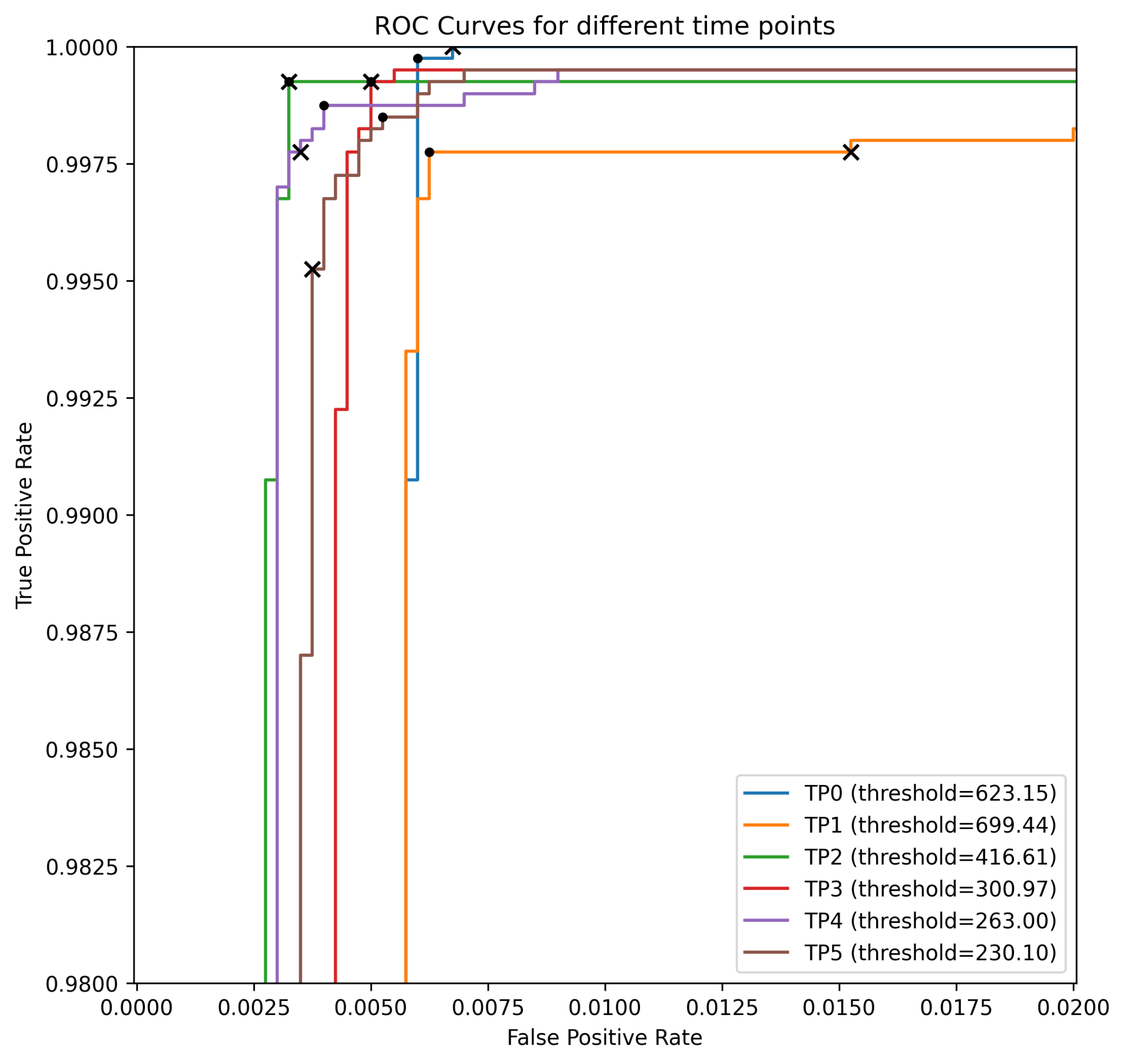


**Figure S9. Species competition - ROC curves for *Escherichia coli* stained on time points TP0-TP5.** Based on a ROC curve at each time point a separate threshold was determined for the classification between stained Ec and unstained Pf (monocultures). The optimal threshold for each time point (black dots) is plotted next to the general threshold (black cross) on each ROC curve. Colours represent time points for which the ROC was drawn. The general threshold is very close to the optimal threshold at each time point. Note: x- and y-axis are zoomed in to resolve minute differences in ROC curves.

**Figure S10. Genotype competition - ROC curves for I7 stained on time points TP0-TP5.** Based on a ROC curve at each time point a separate threshold was determined for the classification between stained I7 and unstained I6 (monocultures). The optimal threshold for each time point (black dots) is plotted next to the general threshold (black cross) on each ROC curve. Colours represent time points for which the ROC was drawn. The general threshold is very close to the optimal threshold at each time point.
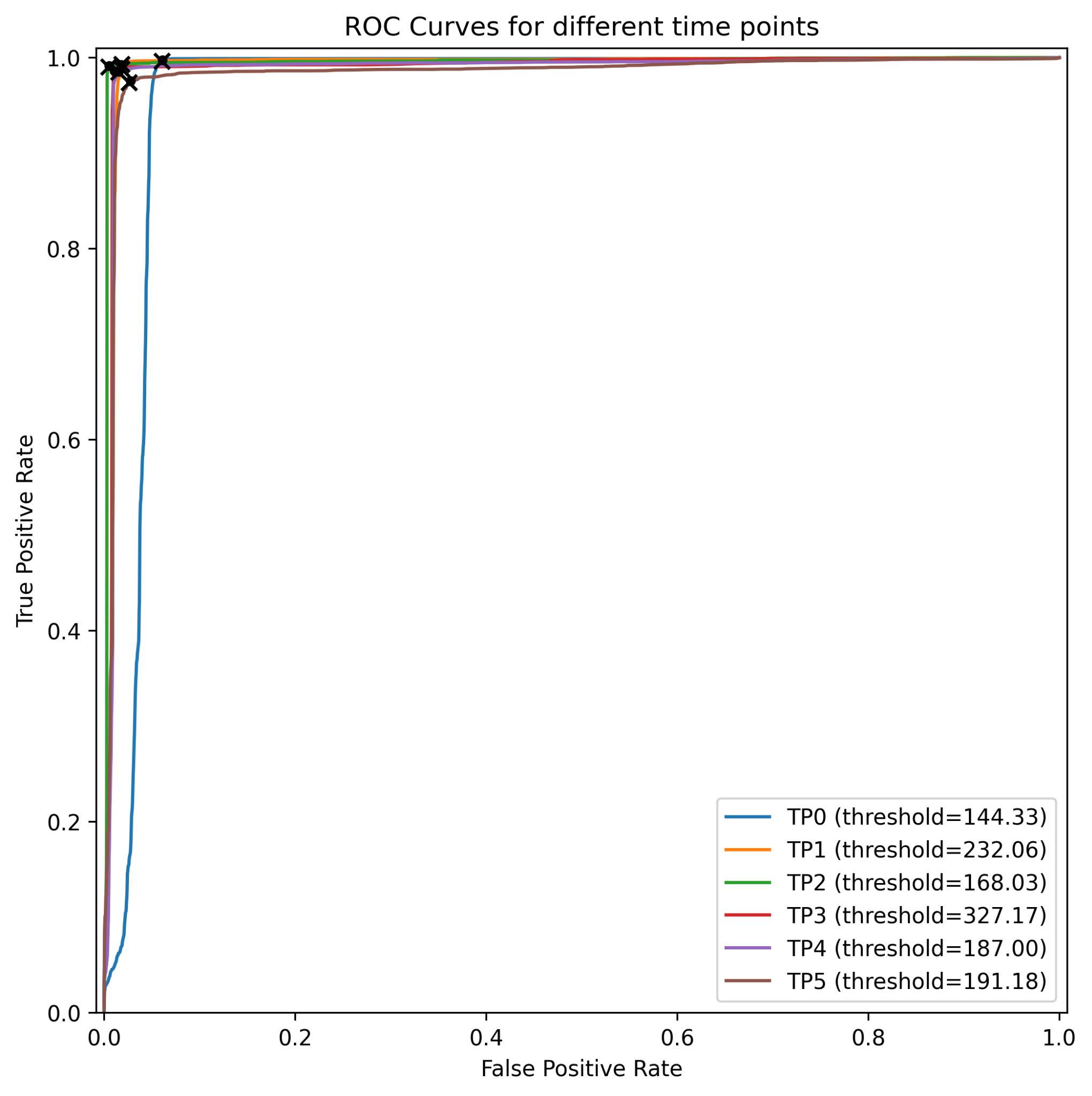


**Figure S11. Genotype competition - ROC curves for I6 stained on time points TP0-TP5.** Based on a ROC curve at each time point a separate threshold was determined for the classification between stained I6 and unstained I7 (monocultures). The optimal threshold for each time point (black dots) is plotted next to the general threshold (black cross) on each ROC curve. Colours represent time points for which the ROC was drawn. The general threshold is very close to the optimal threshold at each time point.
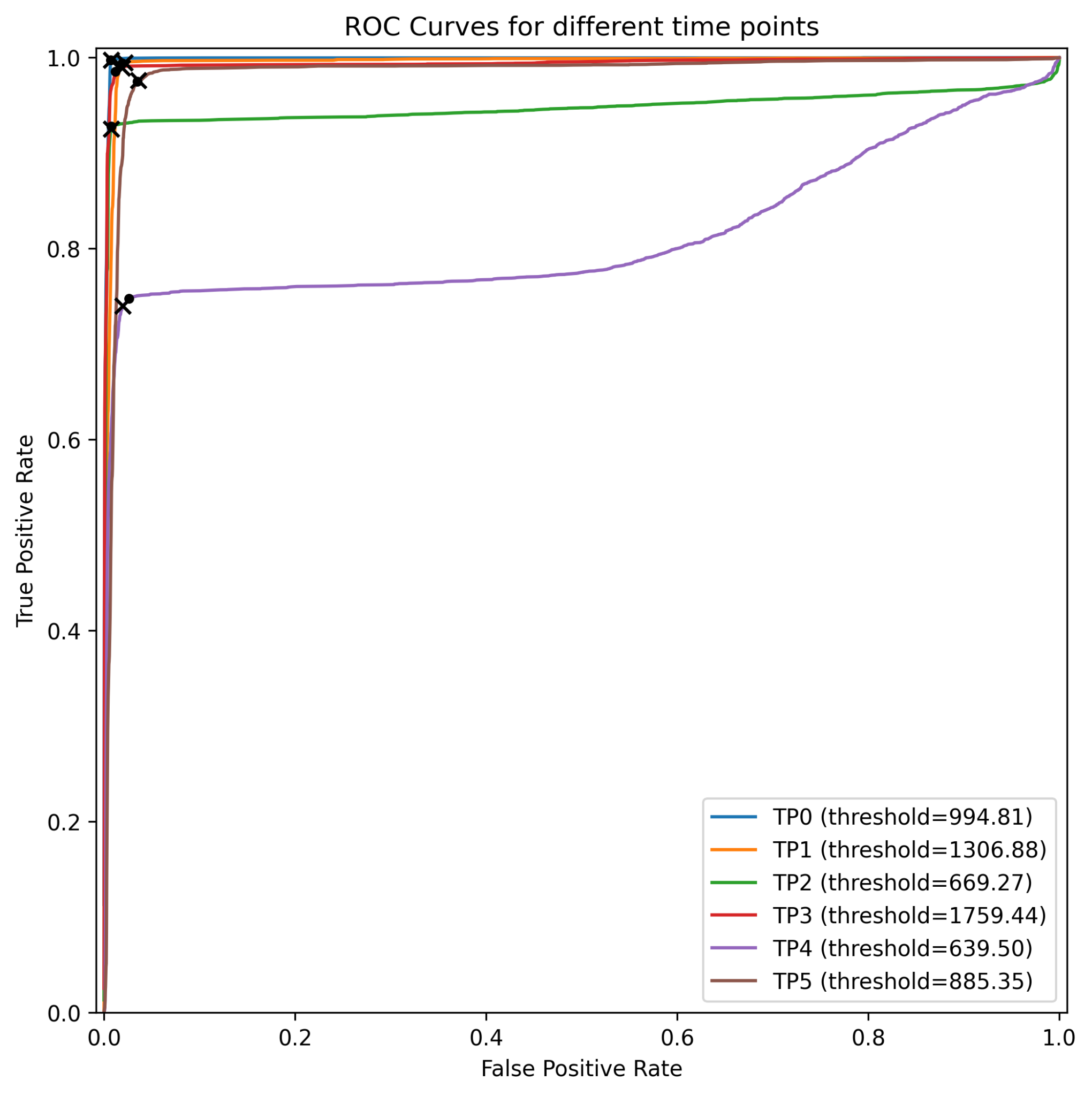


Supplementary Tables

| **Table S1.** Fluorescence thresholds and their performance regarding the un/stained classification are given. Initially, three thresholds (one based on all time points and each one based on TP0 or TP5) were determined for each of the four dye volumes (100µl, 200µl, 300µl and 400µl). The average threshold across dye volumes was calculated for each of the three time points and are displayed in the table. Likewise, performance of the thresholds was evaluated. Average_TPR_TNR is the mean between the True Positive Ratio and the True Negative Ratio. This metric indicates threshold performance on data acquired at the same time point at which the threshold was determined (e.g. threshold based on TP0 data and tested on TP0 data) and shows whether the threshold is well defined on its own time point. Accuracy is the ratio of cells that were correctly classified when the threshold was applied on the entire data set (e.g. threshold based on TP0 data and tested on TP0-TP5 data), thus indicating global threshold performance. | | | | | | |  |
| --- | --- | --- | --- | --- | --- | --- | --- |
| **Time point** | | **threshold** | **averageTPR_TNR** | | | **accuracy** |  |
| TP0 | | 357.083 | 0.993 | | | 0.581 |  |
| TP5 | | 435.651 | 0.992 | | | 0.584 |  |
| all | | 341.899 | 0.993 | | | 0.993 |  |
|  | |  |  | | |  |  |
| **Table S2.** Fluorescence thresholds and their performance regarding the un/stained classification are given. For each of the four dye volumes (100µl, 200µl, 300µl and 400µl) a threshold based on all time points was determined. Performance of the thresholds was evaluated based on average_TPR_TNR (the mean between the True Positive Ratio and the True Negative Ratio) and accuracy (the ratio of cells that were correctly classified). | | | | | | | |
| **dye volume** | **threshold** | | | **average_TPR_TNR** | **accuracy** | | |
| 100 | 256.136 | | | 0.992 | 0.991 | | |
| 200 | 344.889 | | | 0.994 | 0.994 | | |
| 300 | 390.655 | | | 0.993 | 0.993 | | |
| 400 | 375.915 | | | 0.995 | 0.995 | | |

| **Table S3.** Mean proportion of stained cells depending on dye volume. Using a fluorescence threshold based on all time points, cells in samples stained with 100µl, 200µl, 300µl or 400µl dye were classified as stained or unstained. Then the mean and standard error (se) across all time points was calculated. | | | |
| --- | --- | --- | --- |
| **Stained genotype** | **dye volume** | **Stained[%]** | **SE** |
| I7 | 100µ | 99.461 | 0.078 |
| I7 | 200µ | 99.678 | 0.044 |
| I7 | 300µ | 99.294 | 0.315 |
| I7 | 400µ | 99.628 | 0.073 |
| I7 | Ctrl_1 | 0.678 | 0.104 |
| I7 | Ctrl_2 | 0.728 | 0.123 |

| **Table S4.** A linear model (LM, stats::lm) was used to perform linear regression between genotype I7 frequency found on growth agar and determined by fluorescence thresholds. Four indepentend regressions were estimated for each treatment combination of stained genotype(isolate) and predator presence. Formular: freq_I7(stained) ~ stained_isolate:predator/freq_I7_plate -1 . The table gives coefficients of this LM. Rows 1-4 give intercepts and rows 5-8 give slopes of the independent regressions. | | | | |
| --- | --- | --- | --- | --- |
| **Coefficient** | **Estimate** | **Std. Error** | **t value** | **Pr(>\|t\|)** |
| stained_isolate6:predatorNO | 0.026 | 0.028 | 0.932 | 0.353 |
| stained_isolate7:predatorNO | 0.065 | 0.028 | 2.313 | 0.022 |
| stained_isolate6:predatorYES | 0.002 | 0.029 | 0.065 | 0.948 |
| stained_isolate7:predatorYES | 0.084 | 0.028 | 2.985 | 0.003 |
| stained_isolate6:predatorNO:freq_I7_plate | 0.927 | 0.054 | 17.018 | 0.000 |
| stained_isolate7:predatorNO:freq_I7_plate | 0.979 | 0.057 | 17.088 | 0.000 |
| stained_isolate6:predatorYES:freq_I7_plate | 0.877 | 0.056 | 15.635 | 0.000 |
| stained_isolate7:predatorYES:freq_I7_plate | 0.785 | 0.057 | 13.887 | 0.000 |

| **Table S5a**. A linear mixed model (LME, lmerTest::lmer) was fitted to test how relative frequency of genotype I7 changes over time during the competition with genotype I6. The treatment variables initial abundance of I7, predator presence and the stained genotype are included as fixed effects. A random intercept is used to account for the three replicates per treatment combination. Formula: freq_I7~ time + starting_freq_I7 + time:starting_freq_I7 + predator + stained_isolate+ time:predator + time:stained_isolate + (1\|replicate_unique). | | | | | |
| --- | --- | --- | --- | --- | --- |
| **Coefficient** | **Estimate** | **Std. Error** | **df** | **t value** | **Pr(>\|t\|)** |
| (Intercept) | 0.030 | 0.019 | 103.158 | 1.546 | 0.125 |
| time | 0.007 | 0.003 | 174.166 | 2.703 | 0.008 |
| starting_freq_I70.5 | 0.329 | 0.020 | 117.350 | 16.111 | 0.000 |
| starting_freq_I70.9 | 0.750 | 0.020 | 117.350 | 36.750 | 0.000 |
| predatorYES | -0.002 | 0.020 | 73.176 | -0.092 | 0.927 |
| stained_isolate7 | 0.141 | 0.020 | 72.637 | 7.021 | 0.000 |
| time:starting_freq_I70.5 | -0.010 | 0.003 | 174.162 | -3.356 | 0.001 |
| time:starting_freq_I70.9 | -0.011 | 0.003 | 174.162 | -3.513 | 0.001 |
| time:predatorYES | -0.006 | 0.002 | 174.155 | -2.485 | 0.014 |
| time:stained_isolate7 | -0.019 | 0.002 | 174.155 | -7.630 | 0.000 |
| predatorYES:stained_isolate7 | -0.006 | 0.022 | 30.128 | -0.267 | 0.791 |

| **Table S5b.** Analysis of variance on the LME that tested how relative frequency of genotype I7 changes over time during the competition with genotype I6. P<0.05 was taken as significance threshold. | | | | | | | |
| --- | --- | --- | --- | --- | --- | --- | --- |
| **Variable** | **Sum Sq** | **Mean Sq** | **NumDF** | **DenDF** | **F value** | **Pr(>F)** |  |
| time | 0.357 | 0.357 | 1 | 174.155 | 93.687 | 0.000 |  |
| starting_freq_I7 | 5.179 | 2.590 | 2 | 117.191 | 678.932 | 0.000 |  |
| predator | 0.000 | 0.000 | 1 | 117.192 | 0.084 | 0.772 |  |
| stained_isolate | 0.261 | 0.261 | 1 | 117.192 | 68.413 | 0.000 |  |
| time:starting_freq_I7 | 0.060 | 0.030 | 2 | 174.155 | 7.874 | 0.001 |  |
| time:predator | 0.024 | 0.024 | 1 | 174.155 | 6.174 | 0.014 |  |
| time:stained_isolate | 0.222 | 0.222 | 1 | 174.155 | 58.217 | 0.000 |  |
| predator:stained_isolate | 0.000 | 0.000 | 1 | 30.128 | 0.071 | 0.791 |  |

| **Table S5c.** Estimated Marginal Means of linear trends (EMM, emmeans::emtrends, emmeans::test) derived from the LME on genotype I7 frequency. Trends were estimated across fixed effects initial abundance of I7, predator presence and stained genotype. | | | | | | | | |
| --- | --- | --- | --- | --- | --- | --- | --- | --- |
| **predator** | **Stained genotype** | **starting_freq_I7** | **trend** | **SE** | **df** | **t.ratio** | **p.value** |  |
| NO | 6 | 0.1 | 0.007 | 0.003 | 174.031 | 2.703 | 0.008 |  |
| YES | 6 | 0.1 | 0.001 | 0.003 | 174.073 | 0.480 | 0.632 |  |
| NO | 7 | 0.1 | -0.011 | 0.003 | 174.010 | -4.122 | 0.000 |  |
| YES | 7 | 0.1 | -0.017 | 0.003 | 174.031 | -6.343 | 0.000 |  |
| NO | 6 | 0.5 | -0.003 | 0.003 | 174.007 | -0.974 | 0.331 |  |
| YES | 6 | 0.5 | -0.009 | 0.003 | 174.018 | -3.196 | 0.002 |  |
| NO | 7 | 0.5 | -0.021 | 0.003 | 174.018 | -7.799 | 0.000 |  |
| YES | 7 | 0.5 | -0.028 | 0.003 | 174.007 | -10.023 | 0.000 |  |
| NO | 6 | 0.9 | -0.003 | 0.003 | 174.007 | -1.145 | 0.254 |  |
| YES | 6 | 0.9 | -0.009 | 0.003 | 174.018 | -3.368 | 0.001 |  |
| NO | 7 | 0.9 | -0.022 | 0.003 | 174.018 | -7.970 | 0.000 |  |
| YES | 7 | 0.9 | -0.028 | 0.003 | 174.007 | -10.194 | 0.000 |  |

| **Table S6a.** Analysis of variance on a linear model testing how carrying capacity in competition co-cultures depended on initial abundance of genotpye I7, predator presence and the stained genotype. Carrying capacity was calculated as the maximum OD measured within a sample. Formular: OD_max ~ starting_freq_I7 * stained_isolate * predator. P<0.05 was taken as significance threshold. | | | | | |
| --- | --- | --- | --- | --- | --- |
| **variable** | **Df** | **Sum Sq** | **Mean Sq** | **F value** | **Pr(>F)** |
| **character** | **integer** | **numeric** | **numeric** | **numeric** | **numeric** |
| starting_freq_I7 | 2 | 0.021 | 0.010 | 53.630 | 0.000 |
| stained_isolate | 1 | 0.010 | 0.010 | 51.746 | 0.000 |
| predator | 1 | 0.000 | 0.000 | 2.282 | 0.144 |
| starting_freq_I7:stained_isolate | 2 | 0.000 | 0.000 | 0.571 | 0.573 |
| starting_freq_I7:predator | 2 | 0.006 | 0.003 | 15.110 | 0.000 |
| stained_isolate:predator | 1 | 0.000 | 0.000 | 0.589 | 0.450 |
| starting_freq_I7:stained_isolate:predator | 2 | 0.000 | 0.000 | 0.604 | 0.555 |
| Residuals | 24 | 0.005 | 0.000 |  |  |
| n: 8 | | | | | |

| **Table S6b.** Estimated marginal means (lmerTest:emmeans) derived from a linear model testing how carrying capacity in competition co-cultures depended on experimental manipulations. EMM were estimated across the three initial abundances of genotype I7, predator presence and the stained genotype. P<0.05 was taken as significance threshold. | | | | | | | |
| --- | --- | --- | --- | --- | --- | --- | --- |
| **starting_freq_I7** | **predator** | **Stained genotype** | **emmean** | **SE** | **df** | **t.ratio** | **p.value** |
| 0.1 | NO | 6 | 0.305 | 0.008 | 24.000 | 37.960 | 0.000 |
| 0.5 | NO | 6 | 0.289 | 0.008 | 24.000 | 36.050 | 0.000 |
| 0.9 | NO | 6 | 0.287 | 0.008 | 24.000 | 35.717 | 0.000 |
| 0.1 | YES | 6 | 0.339 | 0.008 | 24.000 | 42.279 | 0.000 |
| 0.5 | YES | 6 | 0.283 | 0.008 | 24.000 | 35.302 | 0.000 |
| 0.9 | YES | 6 | 0.248 | 0.008 | 24.000 | 30.858 | 0.000 |
| 0.1 | NO | 7 | 0.347 | 0.008 | 24.000 | 43.276 | 0.000 |
| 0.5 | NO | 7 | 0.334 | 0.008 | 24.000 | 41.615 | 0.000 |
| 0.9 | NO | 7 | 0.310 | 0.008 | 24.000 | 38.624 | 0.000 |
| 0.1 | YES | 7 | 0.365 | 0.008 | 24.000 | 45.519 | 0.000 |
| 0.5 | YES | 7 | 0.316 | 0.008 | 24.000 | 39.414 | 0.000 |
| 0.9 | YES | 7 | 0.278 | 0.008 | 24.000 | 34.637 | 0.000 |

| **Table S7.** A linear model (LM, stats::lm) was used to perform linear regression between Pseudomonas fluorescence frequency found on growth agar and determined by fluorescence thresholds. Two independent regressions were estimated for each stained species. Formular: freq_Pf_ISX ~ stained_species/freq_Pf_plate -1 . The table gives coefficients of this LM. Rows 1-2 give intercepts and rows 3-4 give slopes of the independent regressions. | | | | |
| --- | --- | --- | --- | --- |
| **Coefficient** | **Estimate** | **Std. Error** | **t value** | **Pr(>\|t\|)** |
| stained_speciesEc | -0.086 | 0.137 | -0.625 | 0.536 |
| stained_speciesPf | 0.031 | 0.157 | 0.201 | 0.842 |
| stained_speciesEc:freq_Pf_plate | 0.508 | 0.170 | 2.996 | 0.005 |
| stained_speciesPf:freq_Pf_plate | 0.744 | 0.186 | 3.997 | 0.000 |

| **Table S8a.** A linear mixed model (LME, lmerTest::lmer) was fitted to test how relative frequency Pseudomonas fluorescence changes over time during the competition with Escherichia coli. The treatment variables initial abundance of Pseudomonas fluorescence and the stained species are included as fixed effects. A random intercept is used to account for the three replicates per treatment combination. Formula: freq_Pf_ISX~ time + starting_frequency + time:starting_frequency + stained_species + time:stained_species + (1\|replicate_unique). | | | | | |
| --- | --- | --- | --- | --- | --- |
| **Coefficient** | **Estimate** | **Std. Error** | **df** | **t value** | **Pr(>\|t\|)** |
| (Intercept) | -0.100 | 0.044 | 21.200 | -2.290 | 0.032 |
| time | 0.010 | 0.004 | 85.028 | 2.502 | 0.014 |
| starting_frequency0.5 | 0.276 | 0.054 | 21.200 | 5.153 | 0.000 |
| starting_frequency0.9 | 0.727 | 0.054 | 21.204 | 13.579 | 0.000 |
| stained_speciesPf | 0.268 | 0.044 | 21.203 | 6.121 | 0.000 |
| time:starting_frequency0.5 | 0.009 | 0.005 | 85.021 | 1.832 | 0.070 |
| time:starting_frequency0.9 | -0.003 | 0.005 | 85.059 | -0.705 | 0.483 |
| time:stained_speciesPf | 0.005 | 0.004 | 85.047 | 1.341 | 0.184 |

| **Table S8b.** Analysis of variance on the LME that tested how relative frequency of *Pseudomonas fluorescence* changes over time during the competition with Escherichia coli. P<0.05 was taken as a significance threshold. | | | | | | |
| --- | --- | --- | --- | --- | --- | --- |
| **Variable** | **Sum Sq** | **Mean Sq** | **NumDF** | **DenDF** | **F value** | **Pr(>F)** |
| time | 0.242 | 0.242 | 1 | 85.047 | 52.424 | 0.000 |
| starting_frequency | 0.867 | 0.433 | 2 | 21.203 | 93.979 | 0.000 |
| stained_species | 0.173 | 0.173 | 1 | 21.203 | 37.465 | 0.000 |
| time:starting_frequency | 0.031 | 0.016 | 2 | 85.047 | 3.409 | 0.038 |
| time:stained_species | 0.008 | 0.008 | 1 | 85.047 | 1.798 | 0.184 |

| **Table S8c.** Estimated Marginal Means of linear trends (EMM, emmeans::emtrends, emmeans::test) derived from the LME on Pseudomonas fluorescence frequency. Trends were estimated across fixed effects, initial abundance of Pseudomonas fluorescence and stained species. | | | | | | |
| --- | --- | --- | --- | --- | --- | --- |
| **stained_species** | **starting_frequency** | **trend** | **SE** | **df** | **t.ratio** | **p.value** |
| Ec | 0.1 | 0.010 | 0.004 | 85.007 | 2.502 | 0.014 |
| Pf | 0.1 | 0.015 | 0.004 | 85.007 | 3.847 | 0.000 |
| Ec | 0.5 | 0.018 | 0.004 | 85.007 | 4.743 | 0.000 |
| Pf | 0.5 | 0.023 | 0.004 | 85.007 | 6.088 | 0.000 |
| Ec | 0.9 | 0.006 | 0.004 | 85.026 | 1.628 | 0.107 |
| Pf | 0.9 | 0.011 | 0.004 | 85.101 | 2.931 | 0.004 |

| **Table S9a**. Analysis of variance on a linear model testing how carrying capacity in competition co-cultures depended on initial abundance of Pseudomonas fluorescence and the stained species. Carrying capacity was calculated as the maximum OD measured within a sample. Formula: OD_max ~ starting_frequency * stained_species. P<0.05 was taken as a significance threshold. | | | | | |
| --- | --- | --- | --- | --- | --- |
| **variable** | **Df** | **Sum Sq** | **Mean Sq** | **F value** | **Pr(>F)** |
| **character** | **integer** | **numeric** | **numeric** | **numeric** | **numeric** |
| starting_frequency | 2 | 0.006 | 0.003 | 82.079 | 0.000 |
| stained_species | 1 | 0.000 | 0.000 | 3.663 | 0.080 |
| starting_frequency:stained_species | 2 | 0.000 | 0.000 | 4.521 | 0.034 |
| Residuals | 12 | 0.000 | 0.000 |  |  |
| n: 4 | | | | | |

| **Table S9b.** Estimated marginal means (lmerTest:emmeans) derived from a linear model testing how carrying capacity in competition co-cultures depended on experimental manipulations. EMMs were estimated across the three initial abundances of Pseudomonas fluorescens and the stained species. P<0.05 was taken as a significance threshold. | | | | | | |
| --- | --- | --- | --- | --- | --- | --- |
| **starting_frequency** | **stained_species** | **emmean** | **SE** | **df** | **t.ratio** | **p.value** |
| 0.1 | Ec | 0.076 | 0.003 | 12.000 | 22.171 | 0.000 |
| 0.5 | Ec | 0.049 | 0.003 | 12.000 | 14.357 | 0.000 |
| 0.9 | Ec | 0.045 | 0.003 | 12.000 | 13.087 | 0.000 |
| 0.1 | Pf | 0.092 | 0.003 | 12.000 | 27.054 | 0.000 |
| 0.5 | Pf | 0.052 | 0.003 | 12.000 | 15.138 | 0.000 |
| 0.9 | Pf | 0.041 | 0.003 | 12.000 | 12.111 | 0.000 |

| **Table S10.** Growth parameters estimated for stained and unstained monocultures of genotypes I6 and I7. Parameters were estimated based on two replicates per staining condition using 4th degree polynomal functions via the R package ipolygrowth. | | | | | | |
| --- | --- | --- | --- | --- | --- | --- |
| **staining condition** | **peak growth rate** | **peak growth time** | **doubling time** | **lag time** | **max y** | **max y time** |
| I6 stained | 0.067 | 2.317 | 10.274 | 1.588 | 0.315 | 4.000 |
| I6 unstained | 0.097 | 2.539 | 7.151 | 2.005 | 0.343 | 4.000 |
| I7 stained | 0.073 | 2.444 | 9.555 | 2.006 | 0.284 | 4.000 |
| I7 unstained | 0.063 | 2.295 | 10.982 | 1.774 | 0.280 | 4.000 |

| **Table S11.** Growth parameters estimated for stained and unstained monocultures of the species *E.coli* and *P.fluorescens*. Parameters were estimated based on two replicates per staining condition using 4th degree polynomal functions via the R package ipolygrowth. | | | | | | |
| --- | --- | --- | --- | --- | --- | --- |
| **staining condition** | **peak growth rate** | **peak growth time** | **doubling time** | **lag time** | **max y** | **max y time** |
| Ec stained | 0.018 | 3.452 | 38.850 | 2.640 | 0.084 | 8.000 |
| Ec unstained | 0.027 | 3.239 | 25.490 | 2.690 | 0.101 | 6.000 |
| Pf stained | 0.005 | 2.988 | 141.360 | 2.370 | 0.039 | 10.000 |
| Pf unstained | 0.003 | 2.891 | 255.563 | 1.398 | 0.035 | 10.000 |

**Table S12.** Cell labelling with transient dyes compared to sequencing-based methods and genetic labelling.

| **Feature** | **Genetic Labeling** | **Sequencing-Based** | **Transient Dye Staining (This Study)** |
| --- | --- | --- | --- |
| **Genetic modification required?** | Yes | No | No |
| **Prior genomic information needed?** | No (but useful for marker choice) | Yes (for primer or barcode design) | No |
| **Typical cost per sample** | High (cloning, selective media) | High (library prep, sequencing, bioinformatics) | Low (dye + flow cytometry) |
| **Scalability / throughput** | Low–moderate | High (complex communities) | High but only pairwise |
| **Labor intensity** | High (cloning, strain validation) | Moderate–high (DNA extraction, sequencing steps) | Low (simple staining + cytometry) |
| **Risk of fitness impact** | High (metabolic burden of marker expression) | Low (non-invasive) | Moderate (dye effects assessed in this study) |
| **Best suited for** | Stable, genetically engineered strains | Community composition or long-term evolution experiments | Rapid, high-throughput pairwise competition assays with natural or unmodified isolates |
